# Diversity-driven biochemical survey reveals widespread dimerization throughout the rubisco superfamily

**DOI:** 10.1038/s41467-026-73982-5

**Published:** 2026-06-03

**Authors:** Alexander J. Kehl, Leah Taylor-Kearney, Alexander L. Jaffe, Jose Henrique Pereira, Jennifer Lee, Michal Hammel, Lucas M. Waldburger, Caroline Yeow, Luis Valentin-Alvarado, Paul D. Adams, Jillian F. Banfield, Justin B. Siegel, Noam Prywes, Patrick M. Shih

**Affiliations:** 1 Biophysics Graduate Group, University of California, Davis, Davis, CA USA; 2 Environmental Genomics and Systems Biology Division, Lawrence Berkeley National Laboratory, Berkeley, CA 94720 USA; 3 Feedstocks Division, Joint BioEnergy Institute, Emeryville, CA USA; 4 Department of Plant and Microbial Biology, University of California, Berkeley, Berkeley, CA 94720 USA; 5 Department of Earth System Science, Stanford University, Stanford, CA USA; 6 Molecular Biophysics and Integrated Bioimaging Division, Lawrence Berkeley National Laboratory, Berkeley, CA USA; 7 Genome Center, University of California Davis, Davis, CA USA; 8 Department of Bioengineering, University of California, Berkeley, California, USA; 9 Biological Systems & Engineering Division, Lawrence Berkeley National Laboratory, Berkeley, CA 94720 USA; 10 Department of Electrical Engineering and Computer Science, University of California, Berkeley California, USA; 11 Department of Biochemistry and Molecular Biology, Biomedicine Discovery Institute, Monash University, Clayton, Australia; 12 Department of Bioengineering, University of California, Berkeley, CA 94720 USA; 13 Innovative Genomics Institute, University of California, Berkeley, Berkeley, CA USA; 14 Department of Earth and Planetary Science, University of California, Berkeley, Berkeley, CA USA; 15 Department of Environmental Science, Policy, and Management, University of California, Berkeley, Berkeley, CA USA; 16 Department of Chemistry, University of California-Davis, Davis, California, 95616 USA; 17 Department of Biochemistry and Molecular Medicine, University of California-Davis, Davis, California, 95616 USA; 18 Department of Biochemistry, University of Cambridge, Cambridge, UK

**Keywords:** Structural biology, Enzymes, Evolution

## Abstract

Rubisco is the entry point of nearly all organic carbon into the biosphere and is present in all domains of life. Despite its global importance, biochemical studies of this enzyme superfamily have been limited to a relatively narrow set of subclades. Recent advances in metagenomics have dramatically reshaped our understanding of both microbial and rubisco diversity; however, biochemical characterization of these sequences has not kept pace with the exponential growth in sequence data. To better survey the functional and structural diversity of rubisco, we systematically sample and synthesize a library of diverse rubisco sequences with an emphasis on clades that are sparsely represented in the biochemical literature. Our updated phylogenetic analysis reveals that many deep‑branching rubiscos assemble as dimers, supporting a dimeric origin for the superfamily — in contrast to the ecologically dominant hexadecameric form I. Additionally, we discover and structurally characterize an unusually large catalytic subunit among characterized rubiscos, originating from a early-branching subclade with secondary structural elements not present in canonical rubisco architectures.

## Abstract (web summary)

A survey of rubisco, the enzyme behind nearly all carbon fixation on Earth, across understudied branches of its family tree finds all characterized deep-branching versions are dimers and uncovers a distinct functional unit of rubisco.

## Introduction

Ribulose-1,5-bisphosphate carboxylase/oxygenase (rubisco) is the primary carboxylating enzyme on earth^1,2^, facilitating the conversion of atmospheric carbon dioxide (CO_2_) into the building blocks of life in autotrophic organisms like plants, algae, and bacteria^3^. Rubisco evolved at least three billion years ago, and today is found in eukaryotes, bacteria, and archaea^4^. All rubisco sequences form a single clade that is distinct from rubisco-like proteins (RLPs); however, both rubiscos and RLPs share structural folds and a common evolutionary origin^5,6^. It is thought that rubisco carboxylase activity emerged from a different pre-existing catalytic function in an RLP^2^. Throughout its evolution, rubisco has been adapted to function in all three domains of life and in multiple metabolic pathways, only some of which involve contribute meaningfully to net carbon fixation^7^. Nonetheless, the vast majority of studies have focused on characterizing rubiscos from clades that are involved in autotrophy (i.e., form I and form II); yet, this represents only a fraction of the full diversity of rubiscos found across the entire tree of life. Thus, our understanding of rubisco is largely biased and there remains large gaps in our understanding of rubisco diversity as a whole.

Beyond sequence diversity, rubisco has also emerged as a structurally diverse enzyme family that can adopt a wide range of homo- and hetero-oligomeric states. All rubiscos share a common fold with a distinct N-terminal domain followed by a TIM barrel and a short C-terminal region^1^. The fundamental functional unit of rubisco is composed of an antiparallel dimer of two large subunits (RbcL, ~50 kDa) with a C_2_ symmetry axis and two active sites located at the intradimeric interface. Across the tree of rubiscos, a variety of higher-order assemblages of dimers have been found (L_2_)_n_, including L_2_, L_4_, L_6_, L_8_ and L_10_ as well as L_8_S_8_ hetero-oligomeric structures containing the rubisco small subunit^8–10^. Given this structural diversity, the prevalence and phylogenetic distribution of rubisco dimers remain uncertain, largely due to incomplete coverage of quaternary structures across the phylogeny.

Given their roles in photosynthetic organisms, form I and form II rubiscos have been the most extensively sampled. Recent studies have thoroughly examined the biochemical diversity across sequences in these two subclades revealing a wide range of catalytic rates in rubiscos involved in the Calvin-Benson-Bassham (CBB) cycle^11,12^. Moreover, such efforts have revealed that the fastest rubisco enzymes are among the form II clade. Notably, form I and II rubiscos represent only 10.38% of non-redundant rubisco sequence diversity (Table 1). Moreover, 56.8% of sequences are found in regions of the rubisco tree that lack formal clade designation, representing 62.1% of phylogenetic diversity (Table 1). Ultimately, improving our understanding of rubiscos in sparsely covered regions of the phylogeny may provide distinct insights into the evolution of the broader enzyme family, highlighting the need to revisit the classification and naming scheme of underrepresented rubisco subclades.

**Table 1:** A large fraction of rubiscos remain uncharacterized

Form	Sequence diversity (% of total sequences)	Phylogenetic diversity (% of branch length across phylogeny)
Form I (*sensu stricto*)	6.88%	5.85%
Form II	3.50%	2.82%
Form II/III	2.75%	2.18%
Form IIIa	3.73%	3.61%
Form IIIb	17.5%	15.5%
Form IIIc	6.29%	5.87%
Form III-like	2.55%	2.07%
**All others**	**56.8%**	**62.1%**
Total	100%	100%

**Fig. 1:**
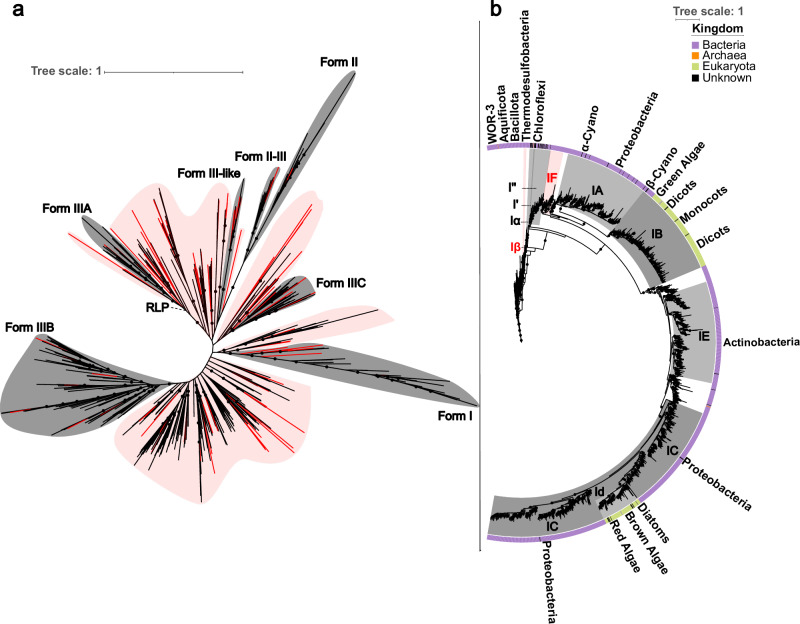
An updated phylogeny reveals many uncharacterized subclades across the rubisco protein family. **a** Phylogenetic tree of the rubisco superfamily. Highlighted in gray are known clades of rubisco. Red branches indicate clusters that are included in our plasmid library. Black dots indicate a bootstrap value of 90% or greater. RLP is shown as a collapsed out-group. Red shading represents areas of the tree that are currently unexplored biochemically. **b** Subtree of form I clade. Highlighted in gray are known subclades of form I. A color bar around the tree indicates the kingdom that the rubisco was found in, purple: Bacteria, orange: Archaea, green: Eukaryota, gray: Unknown. The orange colour is not well visible (zoom in) because only 2 branches have an Archaea lineage host organisms are indicated over the form I tree but are not exhaustive. Black dots indicate a bootstrap value of >90%. Red shading newly defined clades.

Sequence and phylogenetic diversity are measured from a dereplicated phylogeny (1859 clusters) described in Fig. 1 generated from all rubisco sequences in NCBI (~87k). Sequence diversity is measured as the percentage of sequences of each subclade compared to the total number of sequences excluding RLPs (*n* = 435). Phylogenetic diversity is calculated as the sum of the length of all branches, and the percentage of branch length attributable to each subclade. Bold represents areas of the phylogeny that are currently unexplored.

To fill the knowledge gaps in deep-branching, non-CBB associated rubisco sequences across the full phylogeny, we heterologously expressed, systematically synthesized, purified, and carried out biochemical studies on rubiscos from sparsely covered regions and undersampled clades. Our findings clarify the structural origins of the broader rubisco family and reveal secondary structural elements that expand the known structural repertoire of the superfamily within a early-branching clade of exceptionally large rubiscos.

## Results

### Generation of a comprehensive rubisco diversity library

We compiled a comprehensive set of rubisco superfamily protein sequences by querying large databases—specifically NCBI’s non-redundant (nr) and env_nr datasets. Protein sequences were clustered at increasing identity thresholds to account for the high degree of redundancy, particularly within the form I clade. At a 65% identity threshold, we recovered 1859 unique sequence clusters, representing a 14.6% increase over our last effort^1^. Representative sequences were selected from all clusters, regardless of form, and subjected to maximum likelihood phylogenetic reconstruction (Materials and Methods). Across our phylogeny, we estimate that only 8.5% of sequence clusters at 65% identity excluding RLPs have been biochemically characterized for kinetic properties. We utilized our phylogeny to guide the selection of 104 phylogenetically diverse rubisco sequences for biochemical and structural characterization, focusing largely on clades with no previously characterized representatives and/or with evolutionary significance. All 104 genes were synthesized and cloned into an *E. coli* expression plasmid that has previously been used to heterologously express and purify a wide range of rubiscos^8,12^.

### Updated phylogeny resolves form I substructure and uncovers deep-branching clade

Our tree topology of the rubisco superfamily depicts bona fide (i.e., referred to as forms I through III) rubiscos forming a monophyletic clade apart from RLP (Fig. 1a), consistent with previous phylogenetic analyses^1,13,14^. Similarly, we observed that the forms I and II—the only two rubisco forms currently experimentally associated with CBB Cycle—are separated by several short but well-supported internal branches, and do not resolve as sibling clades (Fig. 1a). Within the form I rubiscos, numerous subforms with different taxonomic, structural, and kinetic attributes are currently recognized^1^. Here, we retain the nomenclature for the previously recognized IA, IB, IC, ID and IE subforms (Fig. 1b). However, the phylogenetic relationships between these clades diverge somewhat from previous reconstructions—specifically, we now use form IE to describe a broader, paraphyletic swath of sequences primarily associated with the phylum *Actinobacteria*, overlapping with sequence sets identified in other previous studies^15,16^. On the other hand, we formally classify the merged, monophyletic clade form IF. Finally, we formally classify the clade Iβ.

The revised phylogenetic placement of rubisco sequences provides insight into the evolutionary trajectory of early branching form I sequences. We also observed intriguing taxonomic patterns among sequences placed within our revised form I topology. Notably, rubisco sequences at the deep base of form I—including those from members of the candidate division WOR‑3—derive from bacterial species rather than archaeal ones, distinguishing them from form IIIB; this contrasts with the placement reported by Zeng et al.^17^. These basal bacteria also fall outside the Chloroflexi-associated lineages that dominate later-diverging form I groups, diversifying the evolutionary context from which canonical form Is emerge.

### The majority of deep-branching rubiscos are dimeric

From our synthesized library of 104 diverse rubisco sequences, all were heterologously expressed and purified from *E. coli*, with only 18 that yielded soluble purified enzyme for downstream biochemical characterization and investigation of their oligomeric state. Prior to this study, only four rubiscos have ever been purified from archaea. Here, we purified and characterized seven additional archaeal rubiscos, expanding representation of rubiscos in a poorly sampled domain of life^9,18–20^. Until recently, rubisco dimers were regarded as anomalous within the broader landscape of rubisco oligomerization, primarily associated with the form II clade^8^. However, the discovery of form Iα and I” clades positioned dimeric rubisco basal to the form I lineage, suggesting a dimeric origin for the form I clade^21^. Despite these insights, the prevalence of dimeric rubisco across the full phylogenetic spectrum—particularly among deep-branching clades—remained poorly understood.

**Fig. 2:**
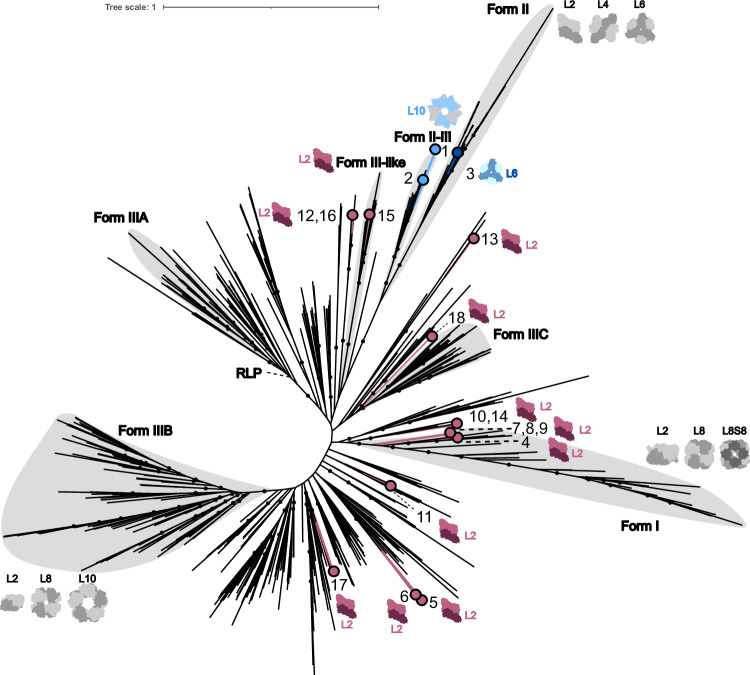
Deep branching rubiscos are dimeric. Phylogenetic tree of rubisco superfamily with oligomeric state of our 18 characterized rubiscos mapped on top. Highlighted in gray are known clades of rubisco. Pink branches indicate clusters that were characterized for oligomeric state. Blue branches indicate clusters that were characterized for oligomeric state in know clades. RLP is shown as a collapsed out group. Rubisco sequences are derived from the following genomes or metagenome assembled genomes. 1: *Candidatus Gottesmaniibacteriota*, 2: *Candidatus Dojkabacteria*, 3: *Rhodopseudomonas faecalis*, 4: *unclassified Bacteria*, 5: *Patescibacteria sp. (*MBU2523915.1*)*, 6: *Patescibacteria sp. (*MBU1445715.1*)*, 7: *Fervidicoccus fontis*, 8: *Thermodesulfobium acidiphilum*, 9: *Ammonifex degensii KC4*, 10: *Aciduliprofundum boonei*, 11: *Altiarchaeales archaeon*, 12: *Lokiarchaeota archaeon*, 13: *unclassified bacteria*, 14: *Methanobacteriota archaeon*, 15: *Kerfeldbacteria bacterium*, 16: *Prometheoarchaeum syntrophicum*, 17: *Candidatus* “Woesearchaeota archaeon CG07_land_8_20_14_0_80_44_2”, 18: *unclassified bacteria*. PDBs used in this figure are as follows form I: 1RBL, 6URA, form II: 5RUB, 5C2C, 7T1C, form IIIB: 1GEH, 2CWX, 8DHT, form II-III: 5MAC, RLP: 2QYG.

To address this gap, we characterized all 18 soluble rubisco variants in our library using Size-exclusion chromatography-small-angle X-ray-scattering (SEC–SAXS). This technique provided bound and unbound scattering profiles for each variant, enabling precise oligomeric state determination (Supplementary Figs. 1, 2). Remarkably, 15 of the 18 rubiscos analyzed—originating from sparsely sampled regions of the phylogenetic—exhibited dimeric quaternary structures (Fig. 2, Supplementary Tables 1, 2). Two of the three variants that exhibited higher-order oligomerization underwent an L2-to-L10 shift upon CABP binding; both are members of the Form II–III clade and contain the RAD domain, which is thought to mediate this oligomeric transition^9^. The remaining variant is a Form II Rubisco that assembles as an L6 oligomer. These diverse sequences underscore the widespread distribution of the dimeric architecture across rubisco phylogeny. Moreover, these findings reinforce the hypothesis that dimerization represents the ancestral state of rubisco.

Taken together, our survey of a 104‑sequence library—yielding 18 soluble enzymes for SEC–SAXS analysis—shows that 15 variants from deep‑branching, sparsely sampled clades adopt dimeric quaternary structures, supporting a dimeric ancestral state and a broader prevalence of dimers than reflected in the existing literature.

### Rubisco genomic context recapitulates major phylogenetic forms

In prokaryotes, rubisco genes are often found in the genome co-locating with a variety of functionally related genes like rubisco activases and other chaperones, regulators, genes involved in the synthesis of carbon concentrating mechanisms, and those involved in the CBB Cycle^22,23^. As an alternative approach to summarizing rubisco diversity and exploring its functional associations, we undertook a genomic context analysis of diverse rubiscos drawn from our non-redundant sequence set. For each rubisco locus with adequate genomic sequence context, we retrieved all protein sequences within 10 open reading frames (ORFs) upstream and downstream, clustered neighboring proteins into families, and then performed multiple dimensionality reduction steps to determine similarities between loci based on their patterns of gene presence/absence (Materials and Methods). Such an approach, previously applied to other microbial genes involved in chlorine cycling^24^, permitted the visualization of 5774 sequence clusters sharing similar (and thus, arguably, functional) context at a high level across phylogeny/taxonomy.

**Fig. 3:**
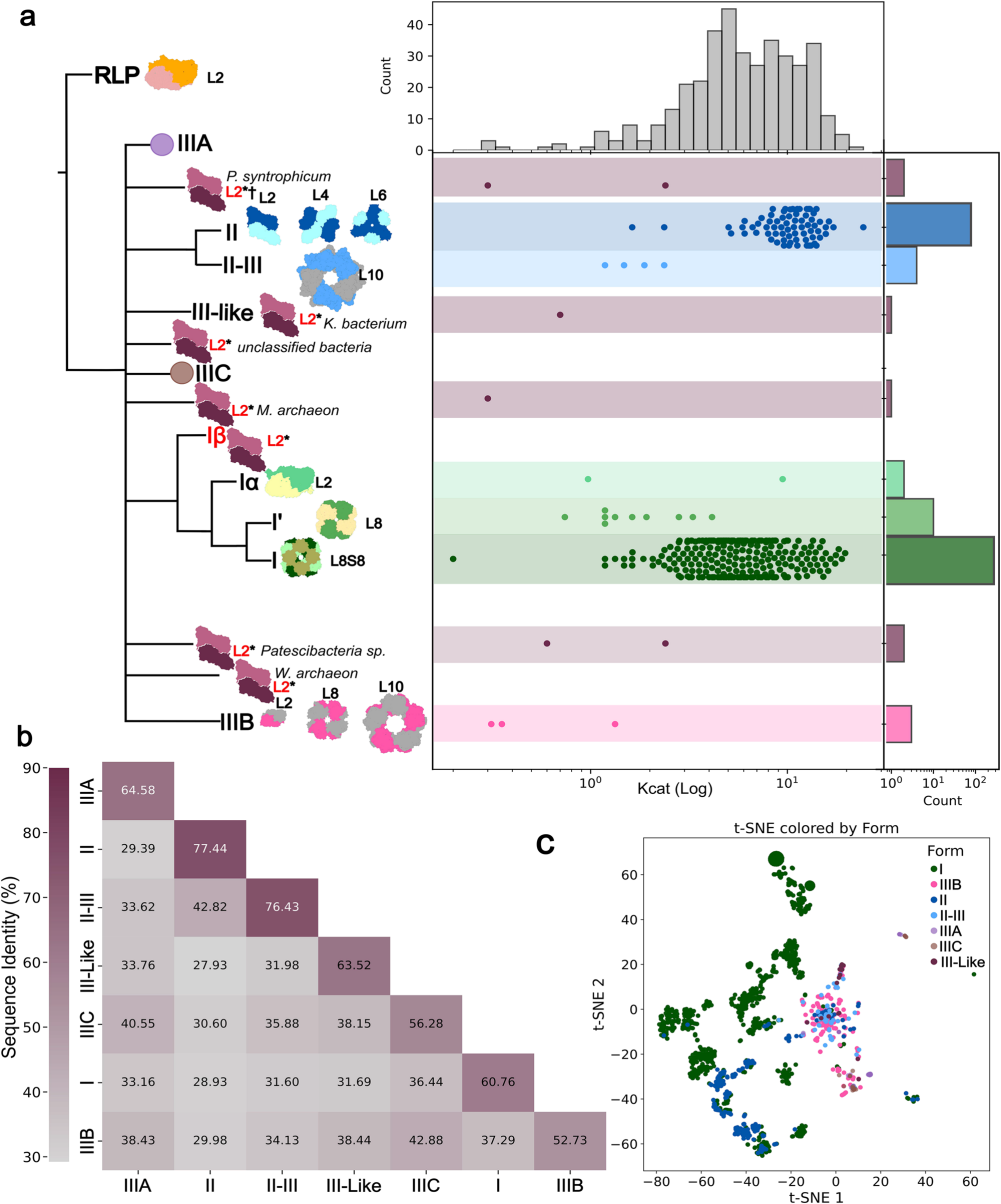
Biochemical, sequence similarity, and genomic context across the rubisco superfamily. **a** Oligomeric states characterized in this study are marked by a * and purple/pink dimer cartoon, † denotes crystal structure presented in this paper, red text denotes rubisco that are in previously unexplored regions of the tree. Clades or clusters with *k*_*catC*_ (Log) measurement in line with the tree. Top histogram shows the count and distribution of *k*_*catC*_(Log) measurements by *k*_*catC*_, the right histogram shows the count and distribution of *k*_*catC*_(Log) measurements by form. **b** Pairwise sequence identity heatmap. Sequence identity is shown for all forms of rubisco with numeric values and color **c:** t-distributed Stochastic Neighbor Embedding (t-SNE) of rubisco sequences colored by form. The following colors are used to depict known clades of rubisco; I: green, IIIB: pink, II: dark blue, II-III: light blue, IIIA: light purple, IIIC: brown, III-Like: dark purple. PDBs used in this figure are as follows form I: 1RBL, 6URA, form II: 5RUB, 5C2C, 7T1C, form IIIB: 1GEH, 2CWX, 8DHT, form II-III: 5MAC, RLP: 2QYG.

Most strikingly, rubisco loci across the tree of life are organized into distinct clusters that align with their phylogenetic form, even though they share some neighboring functional genes. When sampled at a higher identity threshold (95%), we observed numerous form I associated clusters from the IA-IB subclades. However, genomic context could not be reliably retrieved for sequences assigned to the I” clade. (Supplementary Fig. 3c). Form I loci are mostly, though not entirely, distinct from loci with form II rubiscos from bacteria. Loci with form II-III, IIIA, IIIB IIIC, and III-Like formed one large, dense, cluster alongside several smaller ones associated with form II rubiscos (Fig. 3c), suggesting that these rubiscos are likely functionally similar despite substantial phylogenetic divergence (Fig. 3c). Alternatively, divergence from more canonical form I and form II loci could drive clustering of diverse form II-III-Like rubiscos.

Regardless, biochemically characterized rubiscos fell, as expected, primarily within form I clusters (Supplementary Fig. 3b); however, we also observed numerous functional/contextual clusters without any characterized representatives. Among these clusters were those associated with the form I and II but do not encode phosphoribulokinase (*prk*), a key gene that regenerates the rubisco substrate ribulose-1,5-bisphosphate (Supplementary Fig. 4b). While genes encoding the CBB Cycle are known to be spatially dispersed along the chromosome in some organisms – particularly obligate chemoautotrophs that need not tightly regulate the expression of carbon fixation^23^– we nonetheless propose that form I or II sequences in clusters lacking *prk* may standout in terms of their functionality and/or regulatory contexts, and thus should be prioritized for future study. Using comparative genomic context analysis across thousands of rubisco loci, we uncovered distinct, functionally coherent gene neighborhoods that correlate strongly with rubisco form and phylogenetic domain (Fig. 3c).

### Biochemical characterization of phylogenetically diverse rubiscos

To complement our structural analyses, we performed biochemical characterization on a subset of phylogenetically diverse rubiscos. Thermostability (T_m_) measurements were obtained for 10 variants, while catalytic turnover rates (*k*_*catC*_) could only be determined for 7. Thermostability measurements were attempted for all 18 rubiscos; however, sufficient purity was achieved for only the 10 presented here. These sequences span deep-branching regions of the rubisco tree, offering a rare biochemical lens into lineages that remain largely uncharacterized.

Oligomerization has been proposed as an evolutionary strategy for enhancing protein thermal stability^25–27^. To test this hypothesis in the context of rubisco, we surveyed the literature via NCBI PubMed and identified 14 reported T_m_ values: nine from form I rubiscos, one from the form I’ rubisco of *Candidatus Promineofilum breve*, two from form II rubiscos, one from form II-III, and two from the form IIIB rubisco. These measurements are biased toward form I enzymes and were obtained using heterogeneous methods. To broaden the scope of thermal profiling and investigate the structural constraints governing rubisco thermostability, we experimentally determined T_m_ values for 10 phylogenetically diverse rubiscos from our curated library (Table 2). Using a fluorescence-based protein thermal shift assay, we observed melting temperatures ranging from 57.5 °C to 95.2 °C (Table 2, Supplementary Fig. 5).

**Table 2:** Thermal stability of phylogenetically diverse rubiscos

Accession	Organism	form	T_m_ °C	T_m_SD
*Candidatus_Lokiarchaeota_archaeon_strain_HM5_B55*	*Lokiarchaeota archaeon*	unclassified	59.7	0.3
*WP.147662334.1*	*Prometheoarchaeum syntrophicum*	unclassified	61.6	0.4
*MBU1445715.1*	*Patescibacteria sp. (MBU1445715.1)*	unclassified	63.7	0.6
*MBU2523915.1*	*Patescibacteria sp. (MBU2523915.1)*	unclassified	57.5	0.2
*HDR53286.1*	*Methanobacteriota archaeon*	unclassified	78.8	0.1
*MBI5465798.1*	*Kerfeldbacteria sp*.	III-Like	68.7	0
*WP.110780429.1*	*Rhodopseudomonas faecalis*	II	57.6	0.2
*WP_073240254.1*	*Rhodospirillum rubrum*	II	71.8	0.1
*WP.108308545.1*	*Thermosulfobium acidiphilum*	Iβ	77.7	0.4
*PMB76193.1*	*Fervidicoccus fontis*	Iβ	78.3	0.3
*ACX52946.1*	*Ammonifex degensii*	Iβ	95.2	0.2

Previous studies have shown that reducing oligomeric state—by mutating dimer–dimer interface residues in higher-order assemblies (*e.g*., L_10_, L_6_)—can lead to decreased thermal stability, supporting a potential link between oligomerization and thermostability^8,18,27^. However, our data from phylogenetically diverse rubiscos revealed no such correlation between oligomerization and thermostability (Supplementary Fig. 6a). Notably, we identified three dimeric rubiscos within the form Iβ clade that exhibited high T_m_ values ranging from 77.5 °C to 95.2 °C, all derived from thermophilic organisms (Supplementary Fig. 6a). This observation echoes findings from Bundela et al., who reported a dimeric rubisco with the highest known T_m_ (114.5 °C), also from a thermophile^18^. In contrast, we observed a hexameric form II rubisco with a T_m_ of 57.6 °C—comparable to the lowest recorded values (Supplementary Fig. 6a). Taken together, these results suggest that oligomeric state alone does not correlate with thermostability, prompting consideration of alternative factors—such as sequence composition, surface chemistry, or environmental adaptation—that may underlie thermal resilience in rubisco.

To contextualize rubisco’s catalytic constraints, we compared turnover rates across variants characterized in this study, integrating our measurements with recent efforts to expand kinetic profiling beyond historically overrepresented clades (Fig. 3a). Previous studies have highlighted potential sampling bias toward slower, predominantly eukaryotic rubiscos, suggesting that true median activity may be underestimated^12,28,29^. A recent expansion of kinetic measurements across forms I, Iα, II-III, and IIIB supports an updated global median of ≈6 s⁻¹, approaching that of typical enzymes measured at ≈10 s⁻¹^28,30,31^. Yet rubisco remains catalytically constrained relative to central metabolic enzymes, whose *k*_*cat*C_ values often range between 20–80 s⁻¹^30,31^. Our data reinforce this trend while adding kinetic measurements from underrepresented rubisco clades, contributing to a more complete view of rubisco’s catalytic landscape (Supplementary Table 3, Supplementary Fig. 7). Notably, two of the variants we characterized — *Prometheoarchaeum syntrophicum* and *Patescibacteria* sp. (*MBU2523915.1)* — exhibit relatively fast turnover rates (k_catC_ = 2.4 s⁻¹), compared to nearly all deep-branching, non-CBB related rubiscos.

### Large, deep-branching rubisco highlights structural diversity within dimers

**Fig. 4:**
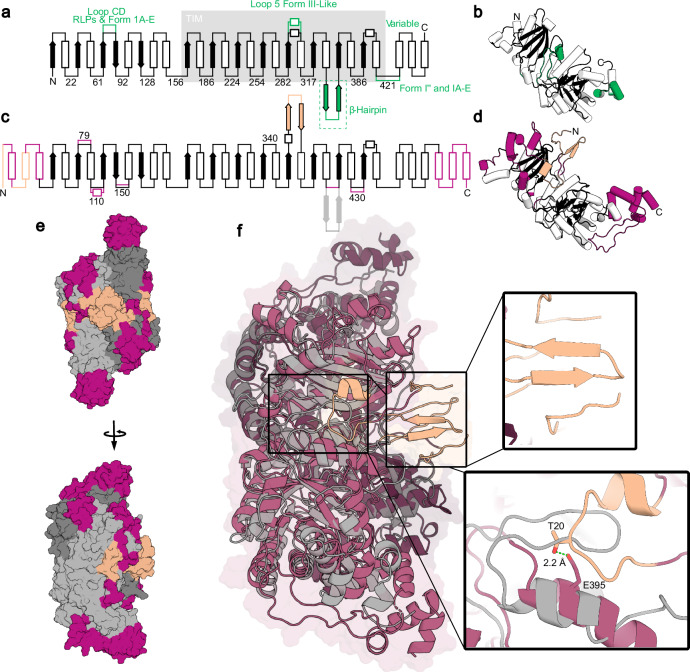
Deep-branching rubisco reveals unprecedented structural diversity of the core rubisco dimer. **a** 2d  topology of rubisco consensus, **b** tertiary structure of consensus rubisco. **c** 2d  topology of *Prometheoarchaeum syntrophicum* rubisco. Insertions shown in purple, with β-sheet and N-terminus shown in orange. **d** Tertiary structure of *Prometheoarchaeum syntrophicum* rubisco, Insertions shown in purple, with β-sheet and N-terminus shown in orange. **e** Surface representation of the crystal structure of *Rhodospirillum rubrum* (9RUB) and apo *Prometheoarchaeum syntrophicum* rubisco (9Z01) superimposed shown in grey, and purple and pink respectively with 90° rotation. Orange as in **d**. **f** Transparent surface and cartoon representation of apo *Prometheoarchaeum syntrophicum* rubisco structure shown in purple and pink, 9RUB shown superimposed in gray, zoom in of distinct β-sheet motif shown in orange, and missing β-hairpin and functional equivalent loop light gray and orange, respectively. 2.2 Å sidechain-sidechain hydrogen bond shown with green dashed line.

Beyond phylogenetic diversity, the expanding catalog of rubisco sequences reveals variants with striking structural features. Two proteins—*L. archaeon* and *P. syntrophicum*—identified in a sparsely sampled deep-branching clade basal to form III-Like, stood out due to their exceptional sequence lengths: 557 and 559 amino acids, respectively. These are approximately 13% longer than other characterized rubisco catalytic subunits in the current literature and 20% larger than the average rubisco length of 466 amino acids (Fig. 4). Their extended sequences include expanded N- and C-termini and multiple insertions, representing primary structure deviations not observed in other characterized rubiscos (Fig. 4a, 4c).

The first insertion, spanning residues 79–84, modestly elongates the loop connecting β-strand 2 to α-helix 5. A second insertion, located at positions 108–124, introduces a short α-helix (helix 6) that bridges α-helix 5 and β-strand 3. Most strikingly, a third insertion between residues 340–354 forms a β-sheet through interaction with the terminal region of the N-terminus—an architecture not observed in canonical rubiscos. To determine the phylogenetic distribution of these motifs, we analyzed the multiple sequence alignment of the 509 rubisco clusters used for our phylogenetic reconstruction (excluding RLPs). We found that the insertions at residues 79–84 and 108–124 are highly restricted, identified in only ~1.6% of sequences and exclusively localized to the *P. syntrophicum* clade. However, the larger insertion at residues 340–354—which forms the β-sheet—is slightly more widespread (~3.9%), appearing in both the *P. syntrophicum* clade and the Form III-like lineage. This suggests that while the β-sheet motif is a conserved structural strategy shared across these deep-branching groups, the additional helical and loop insertions are evolutionary innovations specific to the *P. syntrophicum* lineage. In addition to these insertions, we identified a major deletion (Fig. 4c): the absence of a β-hairpin motif typically conserved in bona fide rubiscos^2^. This hairpin, which extends prominently in forms II-III and III-Like, is notably absent in RLPs and missing in these deep-branching variants.

These changes in primary and secondary structure cascade into the tertiary structure, resulting in visibly distinct monomeric folds compared to the consensus model or any previously characterized rubisco (Fig. 4b, 4d). Together, these structural differences suggest functional divergence and raise intriguing questions about the evolutionary pressures shaping rubisco architecture in these lineages—a hypothesis further supported by the low carboxylation turnover rates observed for this variant.

To explore structure–function relationships in deep‑branching rubiscos, we determined the crystal structure of *P. syntrophicum*, a representative of this unclassified clade with a k_catC_ of 2.4 s⁻¹ (Supplementary Table 4, Supplementary Fig. 8). The structure confirms multiple secondary‑structure motifs observed in the sequence analysis. Most notably, the canonical β‑hairpin motif following loop 6—typically present in bona fide carboxylating rubiscos and essential for active‑site stabilization—is absent. Strikingly, *P. syntrophicum* appears to compensate for this deletion through a previously unobserved interaction: residues 16–26 form an extended loop that engages in a 2.2 Å side‑chain–side‑chain hydrogen bond between T20 and E395 (Fig. 4f). This interaction stabilizes the active‑site region and may substitute mechanically for the missing β‑hairpin. In addition, residues 16–22 contribute hydrophobic packing that mimics the β‑hairpin’s structural support. A further stabilizing element is provided by residues 342–353, which form a β‑sheet that interacts with N‑terminal residues 3–6 via an extended hydrogen‑bonding network (Fig. 4f, Supplementary Fig. 9). This linkage could cascade to stabilize the 16–26 loop, suggesting a convergent strategy for maintaining carboxylation competency.

Relative to *R. rubrum* (Fig. 4e, 4f), a well-characterized form II rubisco, *P. syntrophicum* additionally exhibits an expanded C-terminal region comprising a short terminal α-helix bundle followed by a large unstructured loop spanning residues 502–533. This extension, in combination with the insertions described above, may influence the rigidity of the quaternary structure and, despite being distal from the active sites, could ultimately affect carboxylation efficiency. Phylogenetically, *P. syntrophicum* occupies a deep-branching position within the rubisco clade, and its atypical structural features may reflect early divergence from canonical carboxylation architectures, offering insight into the range of structural plasticity tolerated within the rubisco lineage.

Together, these findings underscore rubisco’s structural plasticity and reveal an alternative architectural solution for preserving carboxylation function in the absence of canonical motifs. The discovery of these compensatory mechanisms shows that rubisco can evolve alternative structural solutions to maintain carboxylation function when canonical motifs are lost and reinforces the need for deeper structural and functional exploration across rubisco clades. Future work to validate the functional role of these structural features will be critical for deepening our understanding of the rubisco structure–function relationship. These motifs may represent an alternative architectural solution and offer an intriguing avenue for future studies into rubisco evolution and engineering.

## Discussion

This study expands the functional and structural characterization of rubisco by exploring under-sampled phylogenetic clades and revising their evolutionary relationships. Rubisco’s catalytic efficiency has long been studied, largely in the context of its importance in photosynthesis and carbon fixation; however, kinetic parameters remain sparsely documented outside form I and form II clades for this reason. To broaden this dataset, we quantified carboxylation turnover (k_cat,C_) for seven deep-branching rubiscos spanning underexplored phylogenetic diversity. Our findings confirm that elevated catalytic rates have evolved only in Form I and II clades, while most rubiscos exhibit slower carboxylation kinetics. A previous study (de Pins et al.) identified one fast (k_cat,C_ > 2 s^−1^) rubisco outside of the form I and form II clades, but upon further analysis we place that enzyme at the base of Form I^28^. This high rate is therefore consistent with an evolution towards faster rates as rubisco became involved in the CBB cycle^32,33^.

This evolutionary drive toward increased efficiency coincided with a fundamental reorganization of the form I clade, which we now show originates from a taxonomically diverse set of basal bacterial lineages. The earliest branching clade within form I, designated Iβ (Fig. 1b, Supplementary Fig. 10), includes genera such as *Ammonifex (Bacillota)*, *Thermodesulfitomonas (Bacillota)*, *Thermodulovibrio (Aquificota)*, and *Thermodesulfobium (Thermodesulfobiota)*^32^. The latter of these was shown to harbor a modified, and perhaps ancestral, form of the CBB cycle^1,32^. This clade bridges the transition from deep-rooted form I sequences with L_2_ dimer oligomerization to those associated with higher-order oligomers L_8_ in form I’ and SSU-containing L_8_S_8_ *Chloroflexi*, and the L_8_S_8_ geometry continues through form IE (predominantly composed of sequences from the *Actinobacteria*) into the IC and IA groups, which are largely composed of *Proteobacteria*. This degree of taxonomic diversity suggests an extensive history of horizontal gene transfer (HGT) involving rubisco, and possibly other components of the CBB pathway, even among a set of relatively late-evolving and structurally constrained rubiscos.

In addition to their phylogenetic significance, the environmental origin of the deep-branching form I groups offer further insights into rubisco diversification. Members of the candidate division WOR-3 inhabit anaerobic sediments, hydrothermal vents, and hot springs, typically under acidic to neutral conditions (pH 2–7) and moderate to high temperatures^17,34^. These organisms are generally facultative anaerobes, engaging in hydrogenotrophic and sulfur-cycling metabolisms^17,34^. In contrast, species within the Iβ clade—*Ammonifex, Thermodesulfitomonas, Thermodulovibrio, and Thermodesulfobium*—are strict anaerobes that thrive in thermophilic, often geothermal or subsurface environments^35–38^. *Ammonifex* and *Thermodesulfobium* are chemolithoautotrophs, utilizing hydrogen and reducing sulfur compounds, while *Thermodesulfitomonas* and *Thermodulovibrio* display organotrophic sulfur-reducing metabolisms^39,40^. Despite broad similarities in temperature and redox conditions, WOR-3’s broader pH tolerance and facultative anaerobiosis distinguish it ecologically from its counterparts with Iβ rubiscos.

These distinct ecological pressures may be drivers of the structural innovations and varied oligomeric states observed across the rubisco superfamily. By mapping these results onto our refined phylogenetic framework alongside our oligomeric state profiling, we demonstrate that the basic dimeric oligomeric state is very widespread, consistent with a dimeric origin of the rubisco superfamily. The observed structural and thermostability diversity, particularly among archaeal sequences, underscores the adaptive breadth of rubisco. In this context, higher‑order assemblies (L₄, L₆, L₈, L₁₀) outside of form I appear to have arisen secondarily, perhaps as adaptive responses to stability demands, with oligomerization emerging as a consequence rather than a primary functional requirement. Such oligomeric complexity may have been driven by the same selective pressures that also shaped the evolution of the small subunit and the recruitment of protein chaperones. Strong selection for catalytic efficiency can destabilize protein structure, fostering alternative folding pathways and promoting interactions with stabilizing partners^41–43^.

The prevalence of dimers among deep‑branching lineages suggests that the ancestral rubisco functioned without the reinforcement of additional subunits. However, once higher-order oligomerization is established, mutations that would be tolerated in dimers become deleterious, preventing a return to the dimeric state and locking in complex architectures^42,44^. Reversion remains possible so long as the interface is stabilized primarily by hydrogen‑bonding networks rather than hydrophobic packing^8^. As Ng et al. point out, the more critical the protein, the more susceptible the organism is to purifying selection of mutations that disrupt the chaperone relationship or, in this case, the higher order oligomerization. Our phylogenetic analysis is consistent with an extension of this hypothesis, since rubiscos involved in the CBB have frequently developed higher order oligomers, it may be the case that the initial establishment of higher order oligomerization or, indeed, chaperone dependence, is more likely when selection is strong and even slight, near-neutral improvements in protein stability come with a strong fitness advantage.

Beyond oligomeric state, deeper structural analysis of deep-branching rubiscos can broaden our understanding of the diversity of secondary structural elements and motifs that have evolved within this enzyme family billions of years ago. Notably, deep-branching forms such as *P. syntrophicum* exhibit atypical secondary structural elements and insertions that expand the known structural repertoire of the enzyme, highlighting a broader range of architectural plasticity and structural diversity than previously recognized. The absence of the β-hairpin in this structure is only seen in forms IIIA and IIIC, as well as all RLPs. The compensation of the β-hairpin in this structure (Fig. 4f) by an extension of Loop 5 raises the question of whether such compensation is necessary for rubisco activity. RLP structures do not compensate in this location (i.e. PDB structures 3QFW or 3FK4). Further structural studies on form IIIA and IIIC may or may not reveal additional compensations testifying to the importance of this structural feature.

Despite being one of the most abundant and consequential enzymes on Earth, rubisco remains unevenly characterized across its vast phylogenetic diversity, with only a fraction of lineages represented by purified and experimentally studied enzymes. This study underscores the difficulty of studying rubiscos from deep branching lineages and archaea; to date, only eleven rubiscos have been purified from an archaeal lineage, seven of which being described in this work. More comprehensive sampling of these clades, which represent the majority of rubisco sequence diversity, may yet reveal surprises. Further sampling at the base of the form I and II branches is also necessary to follow the evolution of rubisco as it became the primary carboxylase on our planet.

## Methods

### Phylogenetic tree inference for the rubisco superfamily

We began by building a comprehensive set of protein sequences from the rubisco superfamily, prioritizing large sequence databases and a subset of publications amalgamating rubisco kinetic data. Specifically, we queried the National Center for Biotechnology Information (NCBI) non-redundant protein database (*nr*) as well as the *env_nr* (November 2024) database using a previously published set of form-specific Hidden Markov Models (HMMs)^45^ using hmmsearch from the HMMER suite^46^. Alignments were filtered to those in which target sequences obtained 90% or greater of overall model coverage. Resulting target sequences were appended to a separate set selected for biochemical characterization in this study, as well as those reported in previous publications^11,12,28,29,47^. To remove partial or composite sequences, the total set was then secondarily filtered to those falling in between 358 and 716 amino acids in length.

Size-filtered sequences were next clustered using USEARCH (*-cluster_fast)* at a variety of identity thresholds. For each cluster at a 65% sequence identity threshold, we selected a representative, prioritizing those which we had attempted biochemical characterization, or, secondarily, those with the largest sequence lengths. Sequences from the Protein Data Bank (PDB) were not selected as representatives due to the frequent appearance of N- and C-terminal expression tags. Similarly, we removed a small number (*n* = 6) of RLP with unstable phylogenetic positions in preliminary reconstructions. We used the same process to more deeply sample the phylogenetic branch including form I rubiscos, this time employing a sequence identity threshold of 90%. Form IIIA rubiscos were used as an outgroup in this case.

For both the full tree and form I subtree, representative sequences were aligned using MAFFT (--auto --reorder)^48^, and alignments were subsequently trimmed using TRIMAL (-gt 0.1)^49^ to remove phylogenetically uninformative positions. Sequence alignments were manually inspected in Geneious. Maximum-likelihood trees were inferred using IQTREE2 with a model selection step and 1000 ultra-fast bootstraps (-bnni, -m TEST -st AA –bb 1000)^50^. Trees were similarly inferred for the four next best evolutionary models, and differences among the five topologies were summarized by calculating the pairwise normalized Robinson-Foulds (nRF) distances (mean nRF = 0.32, σ = 0.01)^51^. Clades were defined through a combination of automated and manual approaches, adjusting clade membership determined in a previous study^1^ in consideration of 1) sequence diversity 2) the degree of bootstrap support 3) degree of taxonomic conservation and 4) prior literature. Characterization status for each representative sequence was assigned based on all members of its corresponding cluster; if any kinetic measurement had been previously made for any cluster member; that cluster was marked as successfully characterized. Similarly, if kinetic measurements had been attempted but failed, we marked clusters accordingly.

### Analysis of genomic context for diverse rubiscos

To reconstruct the genomic loci for diverse rubiscos, we created a more deeply sampled sequence set from the clustering analysis performed above (95% sequence identity). For each of these rubiscos, we queried NCBI using a custom Python script that retrieves all other upstream and downstream protein sequences on the DNA fragment from which the rubisco was originally reported (code available at https://github.com/alexanderjaffe/rubisco-diversity). Taxonomic affiliations for each locus were also obtained through this method. Only loci with at least 10 open reading frames (ORFs) upstream and downstream of the rubisco sequence were retained for further analysis.

Next, all ‘neighbor’ protein sequences were clustered into protein families using MMSeqs (--cov-mode 0)^52^. Protein families were filtered to those with 5 or more members across all rubisco loci, and loci encoding RLPs were removed. Next, we computed the similarity of rubisco loci based on presence/absence patterns of their neighboring genes. This was accomplished by adapting a previous bioinformatic workflow^24^. Briefly, a presence/absence matrix of protein families by locus was created and subjected to Principal Component Analysis (PCA) using Python’s sklearn package. The first two PCA components were then passed to t-distributed stochastic neighbor embedding (t-SNE, also from sklearn) for additional dimensionality reduction. Finally, we visualized loci along the first two t-SNE axes as a function of their rubisco form, taxonomic affiliation, and presence/absence of selected KEGG orthologies (as determined by kofamscan)^53^.

### Plasmids, cloning

Representative rbcL genes were synthesized by JGI (sequences available as supplementary data) and cloned into a pET28 vector with an N-terminal His14-bdSUMO tag Plasmids pSF1389^54^.

### Expression and purification of recombinant proteins

Rubiscos were prepared by cotransforming plasmids containing His14-bdSUMO-tagged rubisco RbcL into chemically competent BL21 DE3 Star *E. coli* with pBADES/EL plasmid. Cells were grown to mid-log phase at 37 °C (OD600  ≈  0.7–0.8) and overexpression of GroEL/ES was induced by the addition of 0.2% w/v arabinose and further incubation for 2 h. Cells were resuspended in fresh TB media (without arabinose) with 100 mM ITPG and shaken for 16 h at 16 °C. Pelleted cells were resuspended in pH 8.0 lysis buffer (20 mM sodium phosphate, 300 mM NaCl, 10 mM imidazole, 5% glycerol, 2 mM MgCl2) with ~5 mM PMSF and subject to a freeze–thaw cycle at −80 °C before lysis by use of a sonicator. The soluble fraction was collected by centrifugation (15,000x g, 20 min). Clarified cell lysate was used to resuspend pre-equilibrated Ni-NTA resin, Ni-NTA resin was allowed to settle before the column was allowed to flow. Columns were washed thoroughly before resuspension in the bdSENP1 reaction buffer (20 mM sodium phosphate pH 8.0, 300 mM NaCl, 10% glycerol). Purified bdSENP1 was added to resuspended columns and rocked gently overnight at 4 °C to facilitate cleavage of the His14-bdSUMO tag from the target protein. Flow-through from the bdSENP1 reaction was collected. Samples were stored in 20 mM sodium phosphate pH 8.0, 150 mM NaCl, 10 mM MgCl2, 10 mM NaHCO3 at −80 °C.

### PAGE analyses

SDS–PAGE samples were prepared according to standard procedures in Laemmli Sample Buffer (Bio-rad) with 2-mercaptoethanol and heated at 98 °C for 5 min. Samples were resolved on 4-12% NuPAGE™ Bis-Tris Mini Protein Gels (Invitrogen) in 1 × Tris/Glycine/SDS buffer (Bio-Rad) and stained in InstantBlue® Coomassie protein stain (Abcam).

### Small-angle X-ray–scattering (SAXS) data collection and analysis

Small-angle X-ray-scattering (SAXS) coupled in line with size-exclusion chromatography (SEC) experiments were performed with 100 µl samples containing 1-45 mg ml^–1^ of rubisco incubated with or without CABP prepared in 20 mM HEPES-OH (pH 8.0), 300 mM NaCl, 10 mM MgCl2, 10 mM NaHCO3. SEC–SAXS data were collected at the ALS beamline 12.3.1 at the Lawrence Berkeley National Laboratory^55^. The X-ray wavelength was set at λ  =  1.127 Å and the sample-to-detector distance was 2,100 mm, resulting in scattering vectors (q) ranging from 0.01 to 0.4 Å−1. The scattering vector is defined as q  =  4πsinθ/λ, where 2θ is the scattering angle. All experiments were performed at 20 °C, and the data were processed as described previously^56^. Briefly, a SAXS flow cell was directly coupled with an inline 1260 Infinity HPLC system (Agilent) using a Shodex KW804 column (Showa Denko). The column was equilibrated with running buffer (20 mM HEPES-OH pH 8.0, 300 mM NaCl, 10 mM MgCl2, 10 mM NaHCO3) with a flow rate of 0.5 ml min–1. A total 90 µl of sample was separated by SEC and 3-s X-ray exposures were collected continuously during a 30-min elution. The SAXS frames recorded before sample analysis were subtracted from all other frames. The subtracted frames were investigated by R_g_ derived by the Guinier approximation, I(q)  =  I(0) exp(–q^2^R_g_2/3) with the limits qR_g_  <  1.6. The elution peak was mapped by comparing the integral of ratios to background and R_g_ relative to the recorded frame using the program RAW^57^. Uniform R_g_ values across an elution peak represent a homogenous assembly. Final merged SAXS profiles, derived by integrating multiple frames across the elution peak, were used for further analysis, including Guinier plot, which determined aggregation-free state. The molecular weight from SAXS was determined by Volume of Correlation (Vc)^58^.

All Size-exclusion chromatography-small-angle X-ray-scattering (SEC-SAXS) data, including processed data, are deposited in the Simple Scattering database (https://simplescattering.com/) with the ID codes listed in the Supplementary Table 1 and Supplementary Table 2.

### SAXS modelling

The atomistic model of rubiscos in the open conformation was prepared based on the crystal structure of the closed conformation presented in this study, with missing N- and C-terminal residues added using the program MODELLER^59^. Different extensions and compactions of the unfolded tails were built to screen conformational variability. The experimental SAXS profiles were then compared to theoretical scattering curves generated from these atomistic models using FoXS^60,61^. Theoretical scattering profiles were fitted to the experimental SAXS data. to validate solution-state conformations of rubiscos. Models of rubisco were obtained through ALPHAFOLD^62^.

### Crystallization and structural determination of rubisco

Ni-NTA–purified rubisco were further subject SEC on a Superose 6 Increase 10/300 GL, in a final buffer containing 100 mM Hepes (pH 8), 100 mM NaCl, 25 mM MgCl2, 5 mM NaHCO3, and 1 mM DTT. The dimeric rubisco *Prometheoarchaeum syntrophicum* (PsRuB) was screened against the following crystallization screens: MCSG-1 (Anatrace); Crystal Screen, SaltRx, PEG/Ion, Index, and PEGRx (Hampton Research); and Berkeley Screen^63^. Crystals of the *Prometheoarchaeum syntrophicum* rubisco were found in 100 mM Bis-Tris-HCl pH 6.5, 100 mM Ammonium phosphate dibasic, 20% (w/v) PEG 3,350 and 5% (v/v/) 2-Propanol. Crystals from the *P. syntrophicum* rubisco were then placed in a reservoir solution containing 20% (v/v) glycerol and flash-cooled in liquid nitrogen.

The X-ray dataset for the PsRuB was collected at the Berkeley Center for Structural Biology beamline 8.2.1 at the Advanced Light Source at Lawrence Berkeley National Laboratory, The diffraction data were processed using the program Xia2^64^. The crystal structure of PsRuB was solved by molecular replacement with the program PHASER^65^ using the search model generated by ALPHAFOLD^62^. The atomic positions obtained from the molecular replacement were used to initiate model building using phenix.autobuild within the Phenix suite^66,67^. Structure refinement was performed using the phenix.refine program^68^. Manual rebuilding was done using COOT^69^. Root mean square deviation differences from ideal geometries for bond lengths, angles, and dihedrals were calculated with Phenix^67^. The stereochemical quality of the final models of *Prometheoarchaeum syntrophicum* were assessed by the program MOLPROBITY^70^. A summary of crystal parameters, data collection, and refinement statistics can be found in Supplementary table 4. Structures and coordinates for PsRuB can be found in the PDB under accession ID 9Z01.

### Rubisco activity assays

The rubisco carboxylation rate was measured using an NADH spectroscopic assay coupled to 3-phosphoglycerate (3-PGA) production, as previously described^12,71,72^. Rubisco was activated in the presence of all assay components minus ribulose-1,5-bisphosphate (RuBP), the substrate. RuBP was synthesised in-house as previously described^73^. Assay activating conditions include; pH 8, 25 °C, 4% CO_2_ and 0.5% O_2_ with orbital shaking at 1440 rpm for ~20 minutes in a 96 well flat-bottom transparent plate (Corning, Costar). The reaction was initiated with the addition of RuBP. NADH oxidation at 340 nm was used to estimate the RuBP turnover rate, accounting for the production of 2 3-PGA molecules per molecule of RuBP. Rubisco active site concentration was estimated using the known rubisco inhibitor, CABP, at *n* = 2, using linear regression (Supplementary Fig. 3). Rates of carboxylation were calculated using methods as previously defined^21^. Two batches of CABP were synthesized from RuBP using both cold and ^14^C-labeled KCN^74,75^. An aliquot of activated *R. rubrum* was used for determination of rubisco active sites via ^14^C-CABP binding and liquid scintillation, as previously described^76^. The ^14^C-CABP quantified *R. rubrum* rubisco was then used to quantify the cold CABP for use in the NADH assay.

### Protein thermal shift (PTS) assay

The PTS assay was conducted using a Protein Thermal Shift kit (Thermo Fisher). Samples were prepared with 1 mg ml^–1^ protein in 1 × PTS phosphate buffer and 4 × PTS dye in Thermo Fisher MicroAmp Optical 8-Tube Strips. The assay was conducted on an Applied Biosciences QuantStudio 3 machine for quantitative PCR with reverse transcription. The assay consisted of initial cooling and hold at 16 °C for 1 min, followed by an 0.05 °C s^–1^ increase to 100 °C and a final hold at 100 °C for 1 min. Data were analyzed in Protein Thermal Shift Software.

### Reporting summary

Further information on research design is available in the Nature Portfolio Reporting Summary linked to this article.

## Supplementary information

Supplementary Information

Description of Additional Supplementary Files

Supplementary Data 1

Supplementary Data 2

Supplementary Data 3

Supplementary Data 4

Supplementary Data 5

Supplementary Data 6

Supplementary Data 7

Supplementary Data 8

Reporting Summary

Transparent Peer Review file

## Data Availability

All data needed to evaluate the conclusions in the paper are present in the paper and/or the Supplementary Materials. Protein sequences used in this study are available in Supplementary Data 7. Data for Phylogenetic trees are provided in Supplementary Data 1 and Supplementary Data 2 respectively. All data for kinetic distribution of the rubisco super family is provided in Supplementary Data 3. All data for pairwise sequence identity plots and TSNE analyses are provided in Supplementary Data 4 and Supplementary Data 5 respectively. All data for sequences represented in this study are provided in Supplementary Data 6. All data for TM analysis are provided in Supplementary Data 8. All Size-exclusion chromatography-small-angle X-ray-scattering (SEC-SAXS) data, including processed data, are deposited in the Simple Scattering database (https://simplescattering.com/) with the ID codes listed in the Supplementary Table 1 and Supplementary Table 2. The structure of PsRub has been deposited in the PDB under accession ID 9Z01. The structures for 3QFW, 1RBL, 6URA, 5RUB, 5C2C, 7T1C, 1GEH, 2CWX, 8DHT, 5MAC, 2QYG, 9RUB and 3FK4 are available in the PDB.

## Code availability

Code produced for this study is available at the following link: https://zenodo.org/records/19614364.
