# Supplementary material for: Diversity-driven biochemical survey reveals widespread dimerization throughout the rubisco superfamily
Source: Nat Commun. 2026 Jun 3;17:8069. doi: 10.1038/s41467-026-73982-5 (PMC13454268; doi:10.1038/s41467-026-73982-5)
Supplement: Supplementary file 1 [file 41467_2026_73982_MOESM11_ESM.pdf]

nature portfolio

Corresponding author(s): Patrick Shih

Last updated by author(s): Apr 29, 2026

Reporting Summary

Nature Portfolio wishes to improve the reproducibility of the work that we publish. This form provides structure for consistency and transparency in reporting. For further information on Nature Portfolio policies, see our Editorial Policies and the Editorial Policy Checklist.

Statistics

For all statistical analyses, confirm that the following items are present in the figure legend, table legend, main text, or Methods section.

n/a	Confirmed	
☐	☒	The exact sample size (*n*) for each experimental group/condition, given as a discrete number and unit of measurement
☒	☐	A statement on whether measurements were taken from distinct samples or whether the same sample was measured repeatedly
☒	☐	The statistical test(s) used AND whether they are one- or two-sided<br>*Only common tests should be described solely by name; describe more complex techniques in the Methods section.*
☒	☐	A description of all covariates tested
☒	☐	A description of any assumptions or corrections, such as tests of normality and adjustment for multiple comparisons
☐	☒	A full description of the statistical parameters including central tendency (e.g. means) or other basic estimates (e.g. regression coefficient) AND variation (e.g. standard deviation) or associated estimates of uncertainty (e.g. confidence intervals)
☒	☐	For null hypothesis testing, the test statistic (e.g. *F*, *t*, *r*) with confidence intervals, effect sizes, degrees of freedom and *P* value noted<br>*Give P values as exact values whenever suitable.*
☒	☐	For Bayesian analysis, information on the choice of priors and Markov chain Monte Carlo settings
☒	☐	For hierarchical and complex designs, identification of the appropriate level for tests and full reporting of outcomes
☒	☐	Estimates of effect sizes (e.g. Cohen's *d*, Pearson's *r*), indicating how they were calculated

Our web collection on statistics for biologists contains articles on many of the points above.

Software and code

Policy information about availability of computer code

Data collection	No previously unreported code or algorithms were used to collect the data in this study.
Data analysis	MODELLER was used to add missing N and C terminals during SAXS analysis, Protein thermal shif data were analysed in Protein Thermal Shift Software, hmmsearch was used to query large sequence databases, USEARCH was used for clustering of sequences, MAFFT was used for sequence alignment, TRIMAL was used to trim sequence alignment, IQTREE2 was used to construct the phylogenetic tree, a custom python script was used to retrieve all other upstream and downstream protein sequences on the DNA fragment from which the rubisco was originally reported (code available at https://github.com/alexanderjaffe/rubisco-diversity). Python's sklearn package was used to to conduct the Principal Component Analysis, The experimental SAXS profiles were then compared to theoretical scattering curves generated from these atomistic models using FoXS, Models of rubisco were obtained through ALPHAFOLD, The diffraction data were processed using the program Xia2, crystal structure of PsRuB was solved by molecular replacement with the program PHASER, atomic positions obtained from the molecular replacement were used to initiate model building using phenix.autobuild within the Phenix suite, Manual rebuilding was done using COOT, Root mean square deviation differences from ideal geometries for bond lengths, angles, and dihedrals were calculated with Phenix, stereochemical quality of the final models of Prometheoarchaeum syntrophicum were assessed by the program MOLPROBITY,

For manuscripts utilizing custom algorithms or software that are central to the research but not yet described in published literature, software must be made available to editors and reviewers. We strongly encourage code deposition in a community repository (e.g. GitHub). See the Nature Portfolio guidelines for submitting code & software for further information.

Data

Policy information about availability of data

All manuscripts must include a data availability statement. This statement should provide the following information, where applicable:

- Accession codes, unique identifiers, or web links for publicly available datasets
- A description of any restrictions on data availability
- For clinical datasets or third party data, please ensure that the statement adheres to our policy

All data needed to evaluate the conclusions in the paper are present in the paper and/or the Supplementary Materials. Protein sequences used in this study are available in SI_data_7_Solubility_Screen. Data for Phylogenetic trees are provided in SI_data_1_full_tree_metadata and SI_data_2_form_1_sub_tree_metadata respectively. All data for kinetic distribution of the rubisco super family is provided in SI_data_3_kinetics_data. All data for pairwise sequence identity plots and TSNE analyses are provided in SI_data_4_pairwise_sequence_identity_matrix and SI_data_5_TSNE_metadata respectively. All data for sequences represented in this study are provided in SI_data_6_Sequence_Library. All data for TM analysis are provided in SI_dataset_8_TM_information. All Size-exclusion chromatography-small-angle X-ray-scattering (SEC-SAXS) data, including processed data, are deposited in the Simple Scattering database (https://simplescattering.com/) with the ID codes listed in the Supplementary Table 1 and Supplementary Table 2. The structure of PsRub has been deposited in the PDB under accession ID 9Z01. The structures for 3QFW, 1RBL, 6URA, 5RUB, 5C2C, 7T1C, 1GEH, 2CWX, 8DHT, 5MAC, 2QYG and 3FK4 are available in the PDB.

Research involving human participants, their data, or biological material

Policy information about studies with human participants or human data. See also policy information about sex, gender (identity/presentation), and sexual orientation and race, ethnicity and racism.

Reporting on sex and gender	Na
Reporting on race, ethnicity, or other socially relevant groupings	Na
Population characteristics	Na
Recruitment	Na
Ethics oversight	Na

Note that full information on the approval of the study protocol must also be provided in the manuscript.

Field-specific reporting

Please select the one below that is the best fit for your research. If you are not sure, read the appropriate sections before making your selection.

☒ Life sciences ☐ Behavioural & social sciences ☐ Ecological, evolutionary & environmental sciences

For a reference copy of the document with all sections, see nature.com/documents/nr-reporting-summary-flat.pdf

Life sciences study design

All studies must disclose on these points even when the disclosure is negative.

Sample size	No statistical analyses were used to pre-determine sample size.
Data exclusions	No data was excluded from the analyses.
Replication	All SEC-SAXS and X-ray crystallography experiments were performed once.
Randomization	Samples were not organized into experimental groups, as individual proteins of interest were studied and characterized.
Blinding	Blinding was not relevant to this study due to the targeted nature of selecting individual proteins of interest for characterization.

Reporting for specific materials, systems and methods

We require information from authors about some types of materials, experimental systems and methods used in many studies. Here, indicate whether each material, system or method listed is relevant to your study. If you are not sure if a list item applies to your research, read the appropriate section before selecting a response.

Materials & experimental systems

n/a	Involved in the study
☒	☐ Antibodies
☒	☐ Eukaryotic cell lines
☒	☐ Palaeontology and archaeology
☒	☐ Animals and other organisms
☒	☐ Clinical data
☒	☐ Dual use research of concern
☒	☐ Plants

Methods

n/a	Involved in the study
☒	☐ ChIP-seq
☒	☐ Flow cytometry
☒	☐ MRI-based neuroimaging

Plants

Seed stocks	Na
Novel plant genotypes	Na
Authentication	Na
