# Supplementary material for: Diversity-driven biochemical survey reveals widespread dimerization throughout the rubisco superfamily
Source: Nat Commun. 2026 Jun 3;17:8069. doi: 10.1038/s41467-026-73982-5 (PMC13454268; doi:10.1038/s41467-026-73982-5)
Supplement: Supplementary file 2 [file 41467_2026_73982_MOESM12_ESM.pdf]

nature portfolio

Peer Review File

Diversity-driven biochemical survey reveals widespread dimerization throughout the rubisco superfamily

Corresponding Author: Dr Patrick Shih

This file contains all reviewer reports in order by version, followed by all author rebuttals in order by version.

Attachments originally included by the reviewers as part of their assessment can be found at the end of this file.

Version 0:

Reviewer comments:

Reviewer #1

(Remarks to the Author)

[Editorial Note: Please also see attachment at end of file]

Kehl et al. present a thorough investigation of Rubisco and Rubisco-like protein sequences to identity and classify deep branching groupings of these sequences. This approach has been conductive numerous times throughout the literature, usually with much smaller sequence sets, but the approach here is comprehensive and well developed. The group is highly skilled in these analyses and the tree matches my knowledge of the sequence relationships between the enzymes presented. Integrated analyses of kinetics, thermostability, and structure demonstrate that the dimeric architecture remains widespread, and functionally competent as a carboxylase in some deep branching clades. From the 102 putative Rubisco sequences examined only 18 could be recombinantly expressed in E. coli. From these 7 could be assigned RuBP carboxylase activity, and only 3 displayed a CO_2 fixation rate greater than 1 s^{-1}.

However, a Rubisco from one of the deep branching groups (the most distant to Form I Rubisco characterized?) displayed a CO_2 fixation rate similar to some plant enzymes. They structurally characterize this enzyme to a high-resolution and resolve several novel secondary structural motifs arising from additional residues compared to other known Rubisco structures. The additional motifs are not interrogated in the context of the activity and it is not clear how widespread they are in nature. The authors call this the largest Rubisco described but it is much smaller than other Rubisco holoenzymes in that it is <150 kDa and many Rubisco are >500 kDa. Rather it is the Rubisco with the largest catalytic subunit, or longest RbcL described. Overall, the new data expands the structure and function understanding of Rubisco substantially and gets us closer to an understanding of how carbon fixation evolved.

In addition, the authors determine the oligomeric state and denaturation temperatures of the soluble enzymes and find these two features poorly correlated. Higher-order oligomerization is proposed to have evolved as an adaptive consequence to maintain L_2 stability rather than as a functional role. This interpretation agrees with existing structure and kinetics studies that do not suggest to me any connection between oligomeric states and activity. Again, more progress is made towards a holistic understanding of Rubisco structure and function.

The authors interpret their data in the context of the new sequence topology of Rubisco determine a dimeric origin of Rubisco. I don’t think anyone would doubt such an origin, as it is already obvious from the corpus of existing knowledge on Rubisco/RLP form and function. The study highlights the extreme amount of sequence diversity within the superfamily. They propose a new naming scheme for the whole family to accommodate this and future work, but I fear this will cause more confusion than it solves. Several concerns, particularly the first major point, should be addressed prior to publication as the analysis may fundamentally undermine the manuscript and I am unable to alleviate the concern easily from the material presented.

Major points:
• It is not overtly clear to me that what is presented as Rubisco are Rubisco at all. I randomly picked some of the sequences

from the attached SI material and searched the genetic context of the genes. In some cases, including for some examples below (see pdf for table), the proposed rbcL gene is immediately preceded by a gene annotated for a S-methyl-5-thioribose-1-phosphate isomerase. This would surely make them RLPs that catalyze enolization reactions of this and similar substrates, as has been biochemically defined in the literature (Singh and Tabita, J. Bacteriol. 2009). The authors have examined the operon context of all the Rubisco in the study, and therefore should use this information to clearly distinguish sequences/groups that are RLPs based on this operon context. Proximity to AMP-phosphorylase and ribose-1,5-bisphosphate isomerase would signal bona fide Rubisco involved in AMP metabolism (Sato et al. Science. 2007; Wrighton et al. ISME. 2016).

• The proposed new naming system is flawed for several reasons. 1) The use of Greek and Latin to distinguish between two very different types looks similar and would be confusing (Ia and Iα for example). 2) The clear distinction that Form I Rubisco have small subunits is very useful and easy to communicate, but would be destroyed by the wholesale addition of many Rubisco that are proposed to be called Form I but lack them entirely. 3) It does not make things any clearer, and due to point 1) less clear, than the naming convention it is intended to replace.

I sympathize with the authors on the challenge of categorizing the Rubisco superfamily, especially because of the diversity they have uncovered and will continue to describe. I do not have a perfect answer, however, I think they have an opportunity here to reclassify things in an organized way. A proposed classification for consideration: I agree with the distinction of RLPs as an RLP group as it is already a familiar nomenclature and distinguishes it from bona fide Rubisco. Form I Rubisco should be classified as containing a small subunit with Greek to denote sufficiently large and distinct groups following with a capitalized letter following the order of discovery and with a characterized representative species to contextualize the grouping. Form IF would make sense as a new group since it is distinct and likely represents early Form I enzymes. Perhaps groups without a representative species could be given a lower case as a proposed group placeholder until characterization is established (i.e Form If as already presented).

This would then make the current Form I’ as Form IIB (or whatever Greek most appropriate based on the publication date and the co-authors combined work on Form II Rubisco), rather than Iβ – grounded by the characterized P. breve. R. rubrum would represent Form IIA, and there is plenty of room to include everything else and future discoveries from C – Z. Form III could follow a similar scheme grounded by M. burtonii and T. kodakarensis in at least two divisions. I suspect the authors will be displeased by this suggestion due to their remarkable discovery and naming of Form I’, but it is the style of formatting that I think would be most widely adopted by the field and with room to grow.

• SEC-SAXS was performed in the presence or absence of CABP, which binds tightly to Rubisco active sites and has been documented to induce oligomerization from dimer (L2) to decamer (L10) (Alonso et al. JBC. 2017). Oligomerization from L2 to L10 was induced for two of the Rubisco in this study when CABP was present, but this not discussed. This is an important finding as the two Rubisco do not appear to contain, as best that I can surmise in the absence of explicit accessions, the domain insertion in RbcL biochemically described to mediate this process (Gunn et al. JBC. 2017). Clarity on this should be provided in the manuscript on the basis of their phylogenetic relationship to M. burtonii Rubisco and the current described mechanism of oligomerization.

Minor points:
• The accession codes for specific proteins described biochemically in the manuscript should be clearly documented in the methods or elsewhere so other researchers can confidently analyze/work with them.

• The phylogenetic context of the novel structural features identified in P. syntrophicum should be outlined. Is this something found in multiple lineages or isolated to a few enzymes in a single group?

• Intro: Paragraph 1. “only some of which involve carbon fixation”. I’m not aware of any Rubisco in pathways that don’t involve carbon fixation, its an intrinsic part of the enzyme’s activity regardless of how minor the carbon fixed might be to the overall metabolism of the cell.

• Intro: Paragraph 2 The last sentence doesn’t make sense to me. RLPs are either the retained ancestral form of proto-Rubisco or functionally diverged from an early proto-Rubisco. All RLPs are dimers and so are many Rubisco and it’s the most simple organization of the functional form – so it ought to be the ancestral structure, unless a functional monomer can be observed. What quaternary structures are incomplete if all possible dimeric organizations have been listed and since those with RbcS are firmly locked as L8S8?

• Intro: Paragraph 5 Here and elsewhere in the manuscript the novel Rubisco is described as having a novel fold. My understanding is that protein domain folds are conserved arrangements of secondary structure. Rubisco has two folded domains an N-terminal domain of a ferredoxin fold and a C-terminal domain of a TIM-barrel fold. Mobile regions, minor insertions and deletions that don’t disturb the broad architecture would not constitute a novel fold, and the superposition of the novel structure on 9RUB shows the overall fold is the same. I would avoid this description and instead use a precise articulation of the novel secondary structural elements.

• Since it is explicitly mentioned in the main text, it would be useful to list the Rubisco that were recombinantly expressed but found to be insoluble in the SI materials so that researchers can avoid these species in future work (see Davidi et al. EMBO. 2020 as an example).

• It is stated that “residues 342–353, which form a β sheet that interacts with N terminal residues 3–6 via an extended

hydrogen bonding network (Figure 4f)" but this interaction is not shown in the zoomed in panel depiction of this region.

• How is the denaturation temperature in SI Fig 5H assigned due to multiple similar peaks? The Tm values of Rubisco from the literature alongside the measurements from this study could be listed in an SI file.

• General formatting. Italicization of species names is absent in some sections (methods). Latin terms such as 'in vitro' and 'bona fide' should follow the journal style and be consistent throughout. Genes should be italicized (i.e. rbcL).

Best Regards,
Robbie Wilson

Reviewer #2

(Remarks to the Author)
In the present manuscript, Kehl and colleagues perform a phylogenetic analysis of rubisco while also surveying the functional and structural diversity of the enzyme in poorly characterised regions of the phylogenetic tree. These authors propose a novel nomenclatural scheme for describing present and future rubiscos. In the later part of this work, 18 rubisco were heterologous expressed and purified in E. coli for structural analysis, with Tm measurements also obtained for 10 and kcatC for 7 of these species, respectively. The key finding of this work is the biochemical characterisation of additional rubisco lineages.
As far as I can see, the methodology appears technically sound, it is written in sufficient detail to replicate these experiments, and the data is analysed carefully. Together, this (and other similar) work is of value to the field and serve an important purpose in helping to bridge the gap between the rapidly expanding catalogue of Rubisco sequences and the comparatively limited characterisation of these enzymes at the biochemical level. However, I outline below a number of comments and concerns that I have associated the novelty of these results, and the structure and accessibility of the manuscript.

Major concerns
The structure of this manuscript appears very confused. The two parts of this work (phylogenetic analysis of rubisco Vs. biochemical characterisation of rubisco) do not appear to fit together in a single cohesive story or body of research. It also appears that the author's attempt to shoehorn in a novel nomenclature for the rubisco family which I am not convinced this is the correct place to achieve this. Instead, this should be better suited to a specific article (e.g., a dedicated perspective article).

I also find the language/arguments used throughout the manuscript to be problematic. In several places, including the title, the authors appear to intentionally frame the narrative as though scientists are currently uncertain about the structural origin of Rubisco. This framing is then used to explicitly suggest that the present work "resolves" or definitively clarifies a long-standing mystery which is not true. This is misleading given the existing body of knowledge on this topic. Multiple lines of evidence all suggest that rubisco evolved from a dimeric origin (e.g., 1. The L2 dimer represents the minimal subunit required for catalysis that is common to all rubiscos. 2. Non-carboxylating Form IV rubisco have a dimeric structure. 3. Rubisco at the base of the Form I L8S8 clade have a dimeric structure revealing a clear evolutionary trajectory toward structural complexity along this lineage). This language needs to be revised. There is general consensus among scientists that rubisco evolved from a dimeric origin. These results presented, from a relatively modest number of extra data points, are confirmatory to the current understanding.

Any phylogeny is a hypothesized series of events inferred from an input sequence dataset. The quality of this hypothesis is affected by the input data, the alignment quality and length, the trimming, the model of sequence evolution etc. Accordingly, every phylogeny that is inferred is unique. The author's need to provide further re-assurances through additional analyses that the phylogeny they provide is robust. E.g., Are the same phylogenies in Figure 1 similarly re-produced with the 10 next best models of sequence evolution from the same data. E.g., How well supported are these clades in the present study compared to in previous studies? E.g., How well supported are these rubisco trees compared to trees inferred from other genes of the same species where possible. These experiments are important.

Full kinetic characterisation of these novel characterised rubisco (Sco, KC, KO etc.) falls within the specific scope of this work and would strengthen these results.

Minor concerns
The manuscript in general requires a thorough edit with a focus on clarity and conciseness. In its current form, the text is repetitive and confusing to read.

In addition to the above, at multiple times throughout the results large paragraphs are added which contain either no or very few results. These paragraphs add unnecessary length to the results and should be better moved to the discussion. Some examples of this are in paragraphs beginning Line 191, 202, 216, 291, 363.

I can not (easily) find the model of sequence evolution used for phylogenetic inference. This is critical and should be explicitly described. As above, similar trees should be re-inferred using a range of methods and the topologies compared.

Despite many words in the Results section dedicated to Tm characterisation and this being one important focus of the manuscriot, none of this data is present in the Main Results but rather in Supplementary Material. To me, this data is important to the narrative and should be moved to the main figures.

“Similarly, we removed a small number (n=6) of RLP with unstable phylogenetic positions in preliminary reconstructions”. Please provide more information? How do you know these are artefactual?

Reviewer #3

(Remarks to the Author)
Review of manuscript NCOMMS-25-93333 "Diversity-driven biochemical survey reveals dimeric structural origin of rubisco"

The manuscript describes a combined phylogenetic, biochemical and structural study of rubisco enzymes not previously characterised. As a background to these studies, advances in metagenomics have resulted in an exponetial growth in sequence data. For rubisco this has led to a situation in which the long-standing classification of rubisco into forms I and II only has clearly become outdated. The recent explosion of completed genomic sequencing projects, from diverse organisms, has led to the finding of additional phylogenetically distinct Rubisco forms, including enzymes from organisms that use alternatives to the Calvin-Bassham-Benson pathway to fix CO_2. In particular, many archaea have been shown to contain a separate class of Rubisco, named form III. The fast development has left a gap in the characterisation of the products of the new sequences. This is certainly true for rubisco and the present study aims to fill this gap.

A library of diverse rubisco sequences were sampled and synthesized with an emphasis on clades that had not previously been characterised. As the title indicates, a conclusion from the resulting analysis points to a dimeric origin for the family of rubisco enzymes. This is against the prevailing assumption of an archeal origin of rubisco, but may not be so surprising, considering that a dimer of L subunits is the minimal catalytic unit, and that there are organisms that carry Rubisco enzymes that occupy an intermediate sequence and structural position between the proteobacterial form II and archaeal form III Rubiscos.
In addition the studies reveal the presence of a rubisco with with a novel structural fold not previously observed. Structural characterisation of this new rubisco from the organism Prometheoarchaeum syntrophicum shows a range of insertions, and a major deletion of a beta-hairpin, which is also absent in the group of Rubisco-Like-Proteins (RLPs). The extended C-terminal region and its role in stabilsation (p. 15) is intriguing and may well influence catalysis through the dynamics of closure of the active site.

Points to consider and if addressed, would strengthen the study.

1. The sequence landscape of rubisco is expanding, and will likely continue to do so. Because of this, there may be a need for a revised nomenclature able to accommodate the emergence of additional clades (in fact there are already indications of a need for revision, see e.g ref 9 of the manuscript). Likely, nobody will miss form designations like I’ and I’’.

The new proposed naming convention is discussed in terms of phylogeny, but how does it relate to catalytic prowess? Regarding the discussion on turnover rates on pages 11-12, maximising kcat may not be the only parameter to consider; with rising atmospheric oxygen levels throughout evolution, rubiscos ability to cope with the competing oxygenation reaction must play a role. Has this been considered?

2. Figure 1a. Dots not well visible, even in a blow-up.

3. Figure 1b. Colours designating kingdoms not well visible (especially orange colour).

4. Page 8, first line: should presumably read “…form IIβ, III, V, VI, and VII?

5. Page 8: SEC-SAXS should be written out in full the first time it appears.

5. Table 2. There is an absence of Form IV clade (formerly RLPs) in the list of proposed sequences. Is this intentional or is it reserved for “RLP” in column 2 (proposed clade name)?

6. Page 9. Regarding structural stability pressures, was rising atmospheric oxygen concentrations considered as a driving force towards structural stability/rigidity, i.e. towards hexadecameric structure? (This is not considering the discussion of thermal stability on page 11).

7. Figure 2. Pink branches not well visible. Maybe increase line width?

8. Figure 3 and legend. t-SNE needs an explanation.

9. Figure 4. The green dashed line is not well visible. Maybe circle it?

10. Legend to Figure 4: “rubrum” write out (Rhodospirillum rubrum).

11. The function of the novel -hairpin/sheet in P. syntrophicum rubisco is intriguing. Are there any indications of a function for this new element?

12. Page 22. Synthesis of ribulose-1,5-bisphosphate refers to reference 74. This is a “nested” reference; the original reference is to Horecker et al. (1956). J . Biol. Chem. 218, 785.

Version 1:

Reviewer comments:

Reviewer #1

(Remarks to the Author)
Our concerns have been addressed in the revisions, Thank you.

Best,
Robbie Wilson and Yunlong Zhao

Reviewer #2

(Remarks to the Author)
The author's have satisfactorily addressed my previous comments. Thanks!

Reviewer #3

(Remarks to the Author)
Review of revised manuscript NCOMMS-25-93333A by Shih et al.

There are still some minor details in need of attention (numbering refers to numbers in my initial review):

3. Figure 1b. Colours designating kingdoms not well visible (especially orange colour).
It still takes high enlargement to trace orange colour. Why not add a comment “(the orange colour is not well visible (zoom in) because only 2 branches have an Archaea lineage)”.

5. Page 8: SEC-SAXS should be written out in full the first time it appears.
The first time SEC-SAXS is mentioned is on bottom of page 9 (line 289 in the revised version of the manuscript). This is where the abbreviation should be explained. In the revised manuscript it is written out on page 21 (line 709) and several places following.

12. Page 22. Synthesis of ribulose-1,5-bisphosphate refers to reference 74. This is a “nested” reference; the original reference is to Horecker et al. (1956). J. Biol. Chem. 218, 785.

Reference to Horecker et al. 1956 should be added to the list of references and should be numbered in the text (ref. nr 74). This means current references should be renumbered in the list of references (->75-78).

Horecker, B. L., Hurwitz, J. & Weissbach, A. The enzymatic synthesis and properties of ribulose 1,5-diphosphate. J. Biol. Chem. 218, 785-794 (1956).

Reviewer #4

(Remarks to the Author)
I co-reviewed this manuscript with one of the reviewers who provided the listed reports. This is part of the Nature Communications initiative to facilitate training in peer review and to provide appropriate recognition for Early Career Researchers who co-review manuscripts.

Open Access This Peer Review File is licensed under a Creative Commons Attribution 4.0 International License, which permits use, sharing, adaptation, distribution and reproduction in any medium or format, as long as you give appropriate credit to the original author(s) and the source, provide a link to the Creative Commons license, and indicate if changes were made.
In cases where reviewers are anonymous, credit should be given to 'Anonymous Referee' and the source.
The images or other third party material in this Peer Review File are included in the article's Creative Commons license, unless indicated otherwise in a credit line to the material. If material is not included in the article's Creative Commons license and your intended use is not permitted by statutory regulation or exceeds the permitted use, you will need to obtain permission directly from the copyright holder.
To view a copy of this license, visit https://creativecommons.org/licenses/by/4.0/

We are grateful to the Reviewers for their comments and suggestions on our manuscript. We are enthusiastic to see that the Reviewers appreciated our findings and our in-depth characterisation of these deep-branching rubiscos.

We have been able to address the Reviewers' comments with expanded discussion and inclusion of relevant background material that strengthens the paper overall. Specifically, we have refined our narrative regarding the dimeric origin of rubisco to better reflect the scientific consensus and have withdrawn the proposed naming convention to focus on our biochemical characterizations. Furthermore, we performed an additional robustness analysis of our phylogenetic trees using the top five BIC-ranked substitution models and integrated thermal stability data into the main results to provide a more cohesive narrative.

We have added new text and SI figures to further clarify some Reviewer points. We believe that the revised manuscript addresses the main points raised by the Reviewers. We have included a point-by-point response to the Reviewers. We thank the reviewers, as the manuscript has improved from these changes.

REVIEWER COMMENTS

Reviewer #1 (Remarks to the Author):

Kehl et al. present a thorough investigation of Rubisco and Rubisco-like protein sequences to identity and classify deep branching groupings of these sequences. This approach has been conductive numerous times throughout the literature, usually with much smaller sequence sets, but the approach here is comprehensive and well developed. The group is highly skilled in these analyses and the tree matches my knowledge of the sequence relationships between the enzymes presented. Integrated analyses of kinetics, thermostability, and structure demonstrate that the dimeric architecture remains widespread, and functionally competent as a carboxylase in some deep branching clades. From the 102 putative Rubisco sequences examined only 18 could be recombinantly expressed in E. coli. From these 7 could be assigned RuBP carboxylase activity, and only 3 displayed a CO2 fixation rate greater than 1 s-1.

However, a Rubisco from one of the deep branching groups (the most distant to Form I Rubisco characterized?) displayed a CO2 fixation rate similar to some plant enzymes. They structurally characterize this enzyme to a high-resolution and resolve several novel secondary structural motifs arising from additional residues compared to other known Rubisco structures. The additional motifs are not interrogated in the context of the activity and it is not clear how widespread they are in nature. The authors call this the largest Rubisco described but it is much smaller than other Rubisco holoenzymes in that it is <150 kDa and many Rubisco are >500 kDa. Rather it is the Rubisco with the largest catalytic subunit, or longest RbcL described. Overall, the new data expands the structure and function understanding of Rubisco substantially and gets us closer to an understanding of how carbon fixation evolved.

We agree with the reviewer that the largest Rubisco is not the most accurate terminologically and will instead refer to it as the largest functional unit of Rubisco, as this more clearly describes the Rubisco at hand.

In addition, the authors determine the oligomeric state and denaturation temperatures of the soluble enzymes and find these two features poorly correlated. Higher-order oligomerization is proposed to have evolved as an adaptive consequence to maintain L2 stability rather than as a functional role. This interpretation agrees with existing structure and kinetics studies that do not suggest to me any connection between oligomeric states and activity. Again, more progress is made towards a holistic understanding of Rubisco structure and function.

The authors interpret their data in the context of the new sequence topology of Rubisco determine a dimeric origin of Rubisco. I don't think anyone would doubt such an origin, as it is already obvious from the corpus of existing knowledge on Rubisco/RLP form and function.
To address the reviewer's comment about the dimeric origin claim, we agree with them and will change the language used to be more reflective of the current knowledge on Rubisco/RLP form and function. Instead of language like "determine a dimeric origin of Rubisco" we will update the title and manuscript to include language like "widespread dimerization found throughout the rubisco superfamily"

The study highlights the extreme amount of sequence diversity within the superfamily. They propose a new naming scheme for the whole family to accommodate this and future work, but I fear this will cause more confusion than it solves. Several concerns, particularly the first major point, should be addressed prior to publication as the analysis may fundamentally undermine the manuscript and I am unable to alleviate the concern easily from the material presented.

Major points:

• It is not overtly clear to me that what is presented as Rubisco are Rubisco at all. I randomly picked some of the sequences from the attached SI material and searched the genetic context of the genes. In some cases, including for some examples below (see pdf for table), the proposed rbcL gene is immediately preceded by a gene annotated for a S-methyl-5-thioribose-1-phosphate isomerase. This would surely make them RLPs that catalyze enolization reactions of this and similar substrates, as has been biochemically defined in the literature (Singh and Tabita, J. Bacteriol. 2009). The authors have examined the operon context of all the Rubisco in the study, and therefore should use this information to clearly distinguish sequences/groups that are RLPs based on this operon context. Proximity to AMP-phosphorylase and ribose-1,5-bisphosphate isomerase would signal bona fide Rubisco involved in AMP metabolism (Sato et al. Science. 2007; Wrighton et al. ISME. 2016).

Protein ID	Species	Proposed Form	Genbank ref	Adjacent gene/protein	Adjacent annotation

NQU77245.1	Candidatus Falkowbacter ia bacterium	VI	JABMQE010000 02 3.1	NQU77244.1	S-methyl-5-thioribose-1-phosphate isomerase
MBI49908 65. 1	Candidatus Gottesmanbac ter ia bacterium	VI	JACRNZ0100000 50 .1	MBI49908 66. 1	Hypothetic al, but closest similarity is Methylthiori bos e-1-phosphat e isomerase
MBI34136 62. 1	Candidatus Aenigmatarc hae ota archaeon	V	JACPZZ0100000 34. 1	MBI34136 61. 1	S-methyl-5-thioribose-1-phosphate isomerase
MFC7204 168 .1	Haloferax namakaokahaiae	V	JBHTAA010000 00 5.1	MFC7204 172 .1	ribose 1,5-bisphosphate isomerase

We thank the reviewer for discovering this unexpected syntenic relationship. It is indeed confusing why these bacterial and archaeal genomes would contain RLP-associated genes near bona fide rubiscos. We have verified that these rubiscos are catalytically competent in principle: Sequences in the table have active site residues present that are notably absent in RLPs. Based on the Rubrum sequence, K168, E194, R288, H321, G223, and S68 are all absent in RLPs but present in the genes listed above. This observation raises several interesting possibilities.

1) These genomes could use bona fide rubiscos to catalyse RLP reactions (i.e. Ashida 2003)
2) Rubisco and RLP activities may be retained in similar genomic loci due to an evolutionary or metabolic relationship
3) Some other metabolic pathway is operating in these genomes aside from the CBB, AMP salvage or methionine salvage.

Given the speculative nature of this discussion, we believe that it is outside of the scope of this manuscript and best saved for more detailed future analysis.

• The proposed new naming system is flawed for several reasons. 1) The use of Greek and Latin to distinguish between two very different types looks similar and would be confusing (Ia and Iα for example). 2) The clear distinction that Form I Rubisco have small subunits is very useful and easy to communicate, but would be destroyed by the wholesale addition of many Rubisco that are proposed to be called Form I but lack them entirely. 3) It does not make things any clearer, and due to point 1) less clear, than the naming convention it is intended to replace.

In order to accommodate the reviewers' concerns, we have withdrawn the proposed naming convention from the manuscript.

I sympathize with the authors on the challenge of categorizing the Rubisco superfamily, especially because of the diversity they have uncovered and will continue to describe. I do not have a perfect answer, however, I think they have an opportunity here to reclassify things in an organized way. A proposed classification for consideration: I agree with the distinction of RLPs as an RLP group as it is already a familiar nomenclature and distinguishes it from bona fide Rubisco. Form I Rubisco should be classified as containing a small subunit with Greek to denote sufficiently large and distinct groups following with a capitalized letter following the order of discovery and with a characterized representative species to contextualize the grouping. Form IF would make sense as a new group since it is distinct and likely represents early Form I enzymes. Perhaps groups without a representative species could be given a lower case as a proposed group placeholder until characterization is established (i.e Form If as already presented).

This would then make the current Form I' as Form IIB (or whatever Greek most appropriate based on the publication date and the co-authors combined work on Form II Rubisco), rather than Iβ – grounded by the characterized P. breve. R. rubrum would represent Form IIA, and there is plenty of room to include everything else and future discoveries from C – Z. Form III could follow a similar scheme grounded by M. burtonii and T. kodakarensis in at least two divisions. I suspect the authors will be displeased by this suggestion due to their remarkable discovery and naming of Form I', but it is the style of formatting that I think would be most widely adopted by the field and with room to grow.

We thank the reviewer for their sympathy and for highlighting the need for reclassification. In order to accommodate the reviewers' concerns, we would like to withdraw the proposed naming convention from the manuscript.

• SEC-SAXS was performed in the presence or absence of CABP, which binds tightly to Rubisco active sites and has been documented to induce oligomerization from dimer (L2) to decamer (L10) (Alonso et al. JBC. 2017). Oligomerization from L2 to L10 was induced for two of the Rubisco in this study when CABP was present, but this not discussed. This is an important finding as the two Rubisco do not appear to contain, as best that I can surmise in the absence of explicit accessions, the domain insertion in RbcL biochemically described to mediate this process (Gunn et al. JBC. 2017). Clarity on this should be provided in the manuscript on the basis of their phylogenetic relationship to M. burtonii Rubisco and the currently described mechanism of oligomerisation.

We thank the reviewer for bringing this to our attention. After reviewing our models and sequence information, we found that the RMSD of models of the functional units are 0.757A (KKP92911.1) and 0.615 (OGG30932.1) as compared to the 5MAC pdb functional unit. The RAD domain is present in structural alignment, and key residues are conserved, such as D366

and D368, D385, W388, R389 in both rubiscos. The %id of KKP92911.1 is 59.2%, OGG30932.1 is 78.6% and are found in the same clade as *M. buttonii*. We will include a brief section in the text that will mention the two rubiscos that change oligomeric state. Based on the above information, we believe the mechanism of higher-order oligomerization to be similar to that of *M. burtonii* Rubisco. The following text was added to the "The majority of deep-branching rubiscos are dimeric" section of the results:

"Two of the three variants that exhibited higher-order oligomerization underwent an L2-to-L10 shift upon CABP binding; both are members of the Form II–III clade and contain the RAD domain, which is thought to mediate this oligomeric transition(Gunn et al. 2017). The remaining variant is a Form II Rubisco that assembles as an L6 oligomer."

Minor points:

• The accession codes for specific proteins described biochemically in the manuscript should be clearly documented in the methods or elsewhere so other researchers can confidently analyze/work with them.

We thank the reviewer for bringing this to our attention. Our goal with the data sets is to make them as easy to use and accessible as possible. Accession codes were added to the SI tables to improve accessibility.

• The phylogenetic context of the novel structural features identified in P. syntrophicum should be outlined. Is this something found in multiple lineages or isolated to a few enzymes in a single group?

We thank the reviewer for this insightful suggestion. To quantitatively assess the phylogenetic distribution of these structural elements, we analyzed the multiple sequence alignment (MSA) of the comprehensive Rubisco dataset used for our phylogeny (clustered at 65% identity).

We found that the insertions at residues 79–84 and 108–124 are highly restricted, occurring in approximately 1.6% of representative sequences and appearing exclusively within the same deep-branching clade as P. syntrophicum. In contrast, the insertion at residues 340–354 is slightly more widespread (~3.9%), appearing in both the *P. syntrophicum* clade and the Form III-like lineage. This analysis confirms that while these features are rare globally, they are conserved structural strategies within these specific deep-branching groups.

We propose to add the following text to the "Large, deep-branching rubisco highlights structural diversity within dimers" section of the results:

To determine the phylogenetic distribution of these novel motifs, we analyzed the multiple sequence alignment of the 509 rubisco clusters used for our phylogenetic reconstruction (excluding RLPs). We found that the insertions at residues 79–84 and 108–124 are highly restricted, identified in only ~1.6% of sequences and exclusively localized to the *P. syntrophicum* clade. However, the larger insertion at residues 340–354—which forms the novel β-sheet—is slightly more widespread (~3.9%), appearing in both the *P. syntrophicum* clade and the Form III-like lineage. This suggests that while the β-sheet motif is a conserved structural strategy

shared across these deep-branching groups, the additional helical and loop insertions are unique evolutionary innovations specific to the *P. syntrophicum* lineage.

• Intro: Paragraph 1. “only some of which involve carbon fixation”. I’m not aware of any Rubisco in pathways that don’t involve carbon fixation, its an intrinsic part of the enzyme’s activity regardless of how minor the carbon fixed might be to the overall metabolism of the cell.
This is a good catch, and we would like to thank the reviewer for bringing this to our attention. We propose to change the text here to “ only some of which contribute meaningfully to net carbon fixation”

• Intro: Paragraph 2 The last sentence doesn’t make sense to me. RLPs are either the retained ancestral form of proto-Rubisco or functionally diverged from an early proto-Rubisco. All RLPs are dimers and so are many Rubisco and it’s the most simple organization of the functional form – so it ought to be the ancestral structure, unless a functional monomer can be observed. What quaternary structures are incomplete if all possible dimeric organizations have been listed and since those with RbcS are firmly locked as L8S8?

We have updated the text to read “ Given this structural diversity, the prevalence and phylogenetic distribution of rubisco dimers remains uncertain, largely due to incomplete coverage of quaternary structures across the phylogeny.”

• Intro: Paragraph 5 Here and elsewhere in the manuscript the novel Rubisco is described as having a novel fold. My understanding is that protein domain folds are conserved arrangements of secondary structure. Rubisco has two folded domains an N-terminal domain of a ferredoxin fold and a C-terminal domain of a TIM-barrel fold. Mobile regions, minor insertions and deletions that don’t disturb the broad architecture would not constitute a novel fold, and the superposition of the novel structure on 9RUB shows the overall fold is the same. I would avoid this description and instead use a precise articulation of the novel secondary structural elements.

We agree with the reviewer and are thankful for catching this mistake in terminology. We will update the manuscript to include the phrase “novel secondary structural elements” instead of novel fold

• Since it is explicitly mentioned in the main text, it would be useful to list the Rubisco that were recombinantly expressed but found to be insoluble in the SI materials so that researchers can avoid these species in future work (see Davidi et al. EMBO. 2020 as an example).
We would like to thank you for bringing this to our attention. This information was added to a new SI dataset called SI_data_7_Solubility_Screen.

• It is stated that “residues 342–353, which form a β sheet that interacts with N terminal residues 3–6 via an extended hydrogen bonding network (Figure 4f)” but this interaction is not shown in the zoomed in panel depiction of this region.

We thank the reviewer for this comment. When preparing this manuscript, we had a draft of the figure that included the hydrogen bond network, but felt that including the bonds made the figure feel crowded and decided not to include them in the final figure. The requested figure has been added to the supplementary information as SI figure 9.

• How is the denaturation temperature in SI Fig 5H assigned due to multiple similar peaks? The Tm values of Rubisco from the literature alongside the measurements from this study could be listed in an SI file.

The denaturation temperature in SI fig 5H is assigned by taking into account the relative abundance of the rubisco from the purified sample with SDS page. The rubisco is the largest band on the SDS page, and from this, we assume the peak from the TM assay with the highest amplitude is the peak that corresponds with rubisco. An additional SI dataset that includes the requested information as SI_dataset_8_TM_information.

• General formatting. Italicization of species names is absent in some sections (methods). Latin terms such as 'in vitro' and 'bona fide' should follow the journal style and be consistent throughout. Genes should be italicized (i.e. rbcL).

We appreciate the reviewer's comment and attention to detail. We have edited the text accordingly.

Best Regards,
Robbie Wilson

Reviewer #2 (Remarks to the Author):

In the present manuscript, Kehl and colleagues perform a phylogenetic analysis of rubisco while also surveying the functional and structural diversity of the enzyme in poorly characterised regions of the phylogenetic tree. These authors propose a novel nomenclatural scheme for describing present and future rubiscos. In the later part of this work, 18 rubisco were heterologous expressed and purified in E. coli for structural analysis, with Tm measurements also obtained for 10 and kcatC for 7 of these species, respectively. The key finding of this work is the biochemical characterisation of additional rubisco lineages.
As far as I can see, the methodology appears technically sound, it is written in sufficient detail to replicate these experiments, and the data is analysed carefully. Together, this (and other similar) work is of value to the field and serve an important purpose in helping to bridge the gap between the rapidly expanding catalogue of Rubisco sequences and the comparatively limited characterisation of these enzymes at the biochemical level. However, I outline below a number of comments and concerns that I have associated the novelty of these results, and the structure and accessibility of the manuscript.

Major concerns

The structure of this manuscript appears very confused. The two parts of this work (phylogenetic analysis of rubisco Vs. biochemical characterisation of rubisco) do not appear to fit together in a single cohesive story or body of research. It also appears that the author's attempt to shoehorn in a novel nomenclature for the rubisco family which I am not convinced this is the correct place to achieve this. Instead, this should be better suited to a specific article (e.g., a dedicated perspective article).

Based on comments from the editor and reviewers, we have made changes to the structure of the manuscript to de-emphasize the naming convention that we had previously proposed. We hope that this will improve the flow of the manuscript, and we look forward to future engagement from the broader community on input on a much needed new nomenclature system that could be published in a future study.

I also find the language/arguments used throughout the manuscript to be problematic. In several places, including the title, the authors appear to intentionally frame the narrative as though scientists are currently uncertain about the structural origin of Rubisco. This framing is then used to explicitly suggest that the present work "resolves" or definitively clarifies a long-standing mystery which is not true. This is misleading given the existing body of knowledge on this topic. Multiple lines of evidence all suggest that rubisco evolved from a dimeric origin (e.g., 1. The L2 dimer represents the minimal subunit required for catalysis that is common to all rubiscos. 2. Non-carboxylating Form IV rubisco have a dimeric structure. 3. Rubisco at the base of the Form I L8S8 clade have a dimeric structure revealing a clear evolutionary trajectory toward structural complexity along this lineage). This language needs to be revised. There is general consensus among scientists that rubisco evolved from a dimeric origin. These results presented, from a relatively modest number of extra data points, are confirmatory to the current understanding.

To address the reviewer's comment about the dimeric origin claim, we agree with them and will change the language used to be more reflective of the current knowledge on Rubisco/RLP form and function. Instead of language like "determine a dimeric origin of Rubisco" we will update the manuscript to include language like "widespread dimerization found throughout the rubisco superfamily"

Any phylogeny is a hypothesized series of events inferred from an input sequence dataset. The quality of this hypothesis is affected by the input data, the alignment quality and length, the trimming, the model of sequence evolution etc. Accordingly, every phylogeny that is inferred is unique. The author's need to provide further re-assurances through additional analyses that the phylogeny they provide is robust. E.g., Are the same phylogenies in Figure 1 similarly re-produced with the 10 next best models of sequence evolution from the same data. E.g., How well supported are these clades in the present study compared to in previous studies? E.g., How well supported are these rubisco trees compared to trees inferred from other genes of the same species where possible. These experiments are important.

We agree with the reviewer about the sensitivity of phylogenetic analyses to parameters and sequence input. To address these concerns, we undertook an additional set of analyses to assess the robustness of the tree topologies inferred for the full rubisco superfamily as well as the subset of sequences in/around the form I rubiscos. As now described in the methods section, a maximum-likelihood model selection step was re-run on our multiple sequence alignments using IQTree2, and the top 5 models (as determined by BIC) were retained. Trees were then inferred for each of these 5 models and compared using normalized Robinson-Foulds (nRF) distances, and we observed high topological consistency across all five models (mean nRF = 0.32 for the full superfamily tree; mean nRF = 0.42 for the Form I subtree), with low standard deviations ($\sigma < 0.02$) indicating that no single substitution model produced an outlier topology.

Finally, we anticipate that the phylogenetic trees made for the rubisco large subunit and other genes are likely to differ substantially due to decoupling of their evolutionary trajectories over time, as well as horizontal gene transfer, which appears to be common across the rubisco tree (Jaffe et al. 2018, *Molecular Biology and Evolution*). Therefore, while we agree this would be an interesting analysis, we do not believe it would serve as a useful benchmark for the robustness of our rubisco tree and would be more appropriately performed in a separate study, where it is certain to yield intriguing evolutionary insights.

Full kinetic characterisation of these novel characterised rubisco (Sco, KC, KO etc.) falls within the specific scope of this work and would strengthen these results.

We thank the reviewer for their suggestion. Given the large number of deep-branching rubiscos that we are characterizing for the very first timel, we believe that further kinetic characterisation is beyond the scope of this work, especially because of our focus on the structural origins of the rubisco protein family. An in depth follow up characterization would involve questions of ecological niche, the evolution of multiple kinetic parameters, the tradeoff relationships between kinetic parameters and the relationship between oligomeric states and kinetic parameters.

Minor concerns

The manuscript in general requires a thorough edit with a focus on clarity and conciseness. In its current form, the text is repetitive and confusing to read.

We will update the manuscript to be clearer and more concise to accommodate the reviewers' concerns

In addition to the above, at multiple times throughout the results large paragraphs are added which contain either no or very few results. These paragraphs add unnecessary length to the results and should be better moved to the discussion. Some examples of this are in paragraphs beginning Line 191, 202, 216, 291, 363.

We thank the reviewer for bringing this to our attention and have adjusted the text accordingly.

I can not (easily) find the model of sequence evolution used for phylogenetic inference. This is critical and should be explicitly described. As above, similar trees should be re-inferred using a range of methods and the topologies compared.

As described above, we undertook an additional set of analyses to assess the robustness of the tree topologies inferred for the full rubisco superfamily as well as the form I subtree. A maximum-likelihood model selection step was re-run on our multiple sequence alignments using IQTree2, and the top 5 models (as determined by BIC) were retained. For the full tree, the Q.pfam+G4 was most favored by BIC, whereas for the subtree, the LG+G4 model was most favored. Trees were then inferred for each of these 5 models and compared. The results of this analysis are now summarised in the figure below.

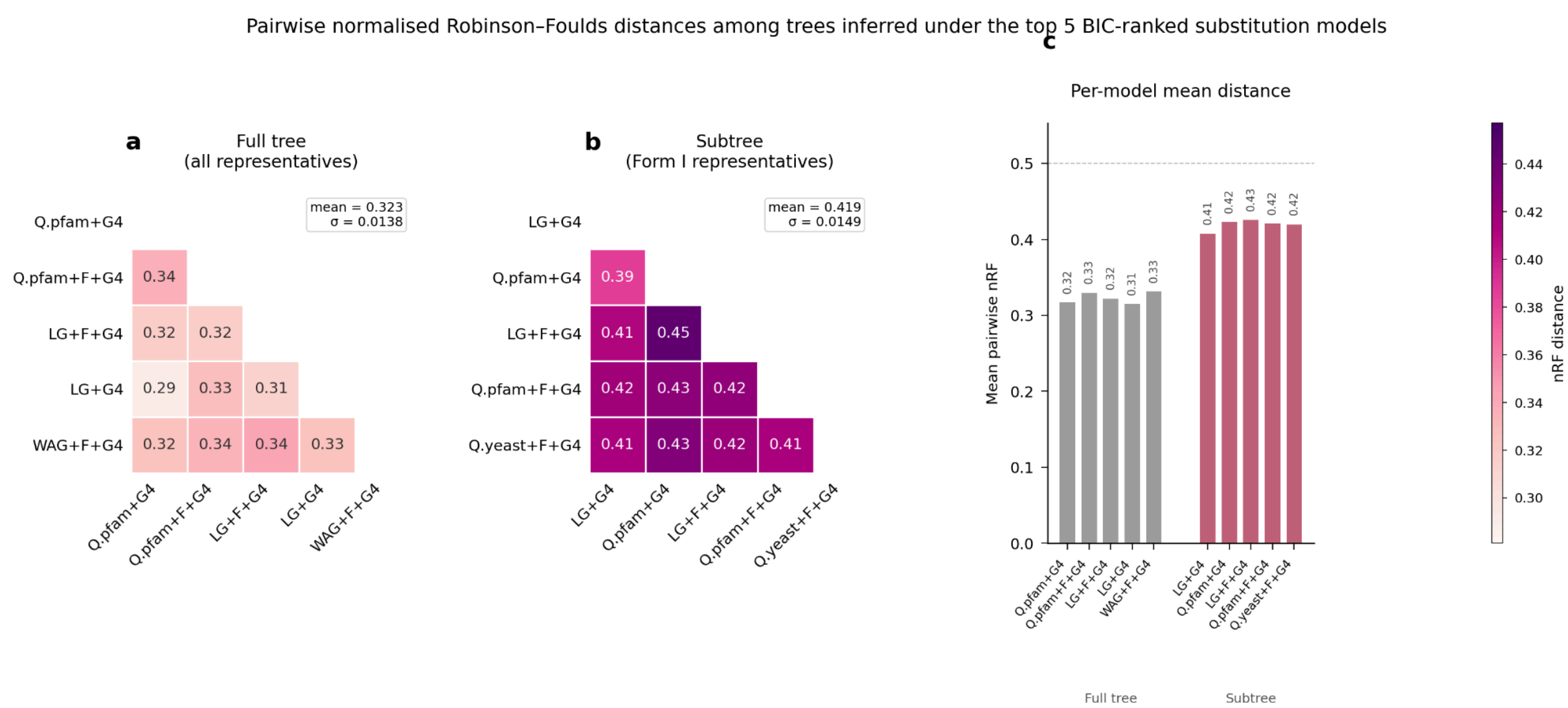

Despite many words in the Results section dedicated to Tm characterisation and this being one important focus of the manuscriot, none of this data is present in the Main Results but rather in Supplementary Material. To me, this data is important to the narrative and should be moved to the main figures.

We would like to thank the reviewer for their insight and have moved TM Table to the main text (Table 2).

“Similarly, we removed a small number (n=6) of RLP with unstable phylogenetic positions in preliminary reconstructions”. Please provide more information? How do you know these are artefactual?

These sequences resolved on very long, individual branches within the *bona fide* rubiscos despite being annotated in NCBI as RLPs. Additionally, the position of these long branches changed substantially each time trees were re-inferred. Thus, we assumed these placements to be artefactual and removed the sequences from our alignments (additionally, RLPs and their internal topology are not a major focus of this study). Such behavior may be observed due to irregularities in sequence composition and/or how they interact with the tree-building algorithm.

Reviewer #3 (Remarks to the Author):

Review of manuscript NCOMMS-25-93333 "Diversity-driven biochemical survey reveals dimeric structural origin of rubisco"

The manuscript describes a combined phylogenetic, biochemical and structural study of rubisco enzymes not previously characterised. As a background to these studies, advances in metagenomics have resulted in an exponetial growth in sequence data. For rubisco this has led to a situation in which the long-standing classification of rubisco into forms I and II only has clearly become outdated. The recent explosion of completed genomic sequencing projects, from diverse organisms, has led to the finding of additional phylogenetically distinct Rubisco forms, including enzymes from organisms that use alternatives to the Calvin-Bassham-Benson pathway to fix CO2. In particular, many archaea have been shown to contain a separate class of Rubisco, named form III. The fast development has left a gap in the characterisation of the products of the new sequences. This is certainly true for rubisco and the present study aims to fill this gap.

A library of diverse rubisco sequences were sampled and synthesized with an emphasis on clades that had not previously been characterised. As the title indicates, a conclusion from the resulting analysis points to a dimeric origin for the family of rubisco enzymes. This is against the prevailing assumption of an archeal origin of rubisco, but may not be so surprising, considering that a dimer of L subunits is the minimal catalytic unit, and that there are organisms that carry Rubisco enzymes that occupy an intermediate sequence and structural position between the proteobacterial form II and archaeal form III Rubiscos.
In addition the studies reveal the presence of a rubisco with with a novel structural fold not previously observed. Structural characterisation of this new rubisco from the organism Prometheoarchaeum syntrophicum shows a range of insertions, and a major deletion of a beta-hairpin, which is also absent in the group of Rubisco-Like-Proteins (RLPs). The extended C-terminal region and its role in stabilsation (p. 15) is intriguing and may well influence catalysis through the dynamics of closure of the active site.

Points to consider and if addressed, would strengthen the study.

1. The sequence landscape of rubisco is expanding, and will likely continue to do so. Because of this, there may be a need for a revised nomenclature able to accommodate the emergence of additional clades (in fact there are already indications of a need for revision, see e.g ref 9 of the manuscript). Likely, nobody will miss form designations like I’ and I’’.

The new proposed naming convention is discussed in terms of phylogeny, but how does it relate to catalytic prowess? Regarding the discussion on turnover rates on pages 11-12, maximising kcat may not be the only parameter to consider; with rising atmospheric oxygen levels

throughout evolution, rubiscos ability to cope with the competing oxygenation reaction must play a role. Has this been considered?
We are thankful for the reviewers' comments and perspective; however, we would like to withdraw the proposed naming convention from the manuscript.

2. Figure 1a. Dots not well visible, even in a blow-up.

The size of the dots in Figure 1a and 1b have been increased.

3. Figure 1b. Colours designating kingdoms not well visible (especially orange colour).

Colours designating kingdoms were made more visible by increasing the saturation of all three colours. The orange colour is hard to see because only 2 branches have an Archaea lineage.

4. Page 8, first line: should presumably read "…form IIβ, III, V, VI, and VII?

Yes, this is a mistake, and we thank the reviewer for noticing.

5. Page 8: SEC-SAXS should be written out in full the first time it appears.

We have updated the text in accordance with this suggestion.

5. Table 2. There is an absence of Form IV clade (formerly RLPs) in the list of proposed sequences. Is this intentional or is it reserved for "RLP" in column 2 (proposed clade name)?

This was a mistake; however, we will be removing the proposed naming convention as requested.

6. Page 9. Regarding structural stability pressures, was rising atmospheric oxygen concentrations considered as a driving force towards structural stability/rigidity, i.e. towards hexadecameric structure? (This is not considering the discussion of thermal stability on page 11).

We did not consider rising atmospheric oxygen concentrations as a diving force towards structural stability/rigidity. This feature was only considered for the kinetic characteristics of the enzymes. Direct evidence to support such a hypothesis can be challenging – e.g., looking for a correlation between SC/O and TM – however, this was not the motivation behind our study and most likely would be better suited for a follow up study.

7. Figure 2. Pink branches not well visible. Maybe increase line width?

We thank the reviewer for this recommendation. The line width of nonblack lines has been increased.

8. Figure 3 and legend. t-SNE needs an explanation.

A more informative explanation has been added.

9. Figure 4. The green dashed line is not well visible. Maybe circle it?

We have edited the zoom-in with the hbond to increase its size and make it more visible.

10. Legend to Figure 4: “rubrum” write out (Rhodospirillum rubrum).

We thank the reviewer for this recommendation. It has been incorporated into the text.

11. The function of the novel -hairpin/sheet in P. syntrophicum rubisco is intriguing. Are there any indications of a function for this new element?

We were curious about this question and performed a comparative structure analysis. We speculate in the text that the novel -hairpin/sheet in P. syntrophicum is helping to stabilize loop 6 in the absence of the β-hairpin motif typically conserved in bona fide rubiscos. Experimental validation of this hypothesis would be challenging and is beyond the scope of this paper.

12. Page 22. Synthesis of ribulose-1,5-bisphosphate refers to reference 74. This is a “nested” reference; the original reference is to Horecker et al. (1956). J . Biol. Chem. 218, 785.

We have updated the text to reflect this correction.

We sincerely thank all reviewers for their time and constructive engagement with our manuscript. Reviewers 1 and 2 confirm that their previous concerns have been fully addressed. Reviewer 3 raised three minor remaining points concerning figure legend clarity, abbreviation placement, and reference formatting, each of which has been resolved as detailed below.

REVIEWERS' COMMENTS

Reviewer #1 (Remarks to the Author):

Our concerns have been addressed in the revisions, Thank you.

Best,
Robbie Wilson and Yunlong Zhao

Reviewer #2 (Remarks to the Author):

The author's have satisfactorily addressed my previous comments. Thanks!

Reviewer #3 (Remarks to the Author):

Review of revised manuscript NCOMMS-25-93333A by Shih et al.

There are still some minor details in need of attention (numbering refers to numbers in my initial review):

3. Figure 1b. Colours designating kingdoms not well visible (especially orange colour).
It still takes high enlargement to trace orange colour. Why not add a comment "(the orange colour is not well visible (zoom in) because only 2 branches have an Archaea lineage)".

We thank the reviewer for this idea, " the orange colour is not well visible (zoom in) because only 2 branches have an Archaea lineage" was added to the figure 1 legend.

5. Page 8: SEC-SAXS should be written out in full the first time it appears.
The first time SEC-SAXS is mentioned is on bottom of page 9 (line 289 in the revised version of the manuscript). This is where the abbreviation should be explained. In the revised manuscript it is written out on page 21 (line 709) and several places following.

SEC-SAXS was written out at line 220, page 6, in the manuscript revision 1, the first time it appears in the manuscript.

12. Page 22. Synthesis of ribulose-1,5-bisphosphate refers to reference 74. This is a "nested" reference; the original reference is to Horecker et al. (1956). J. Biol. Chem. 218, 785.

Reference to Horecker et al. 1956 should be added to the list of references and should be numbered in the text (ref. nr 74). This means current references should be renumbered in the list of references (->75-78).

Horecker, B. L., Hurwitz, J. & Weissbach, A. The enzymatic synthesis and properties of ribulose 1,5-diphosphate. J. Biol. Chem. 218, 785-794 (1956).

Horecker et al. (1956) is currently cited as reference 73 for the synthesis of RuBp.

Reviewer #4 (Remarks to the Author):

I co-reviewed this manuscript with one of the reviewers who provided the listed reports. This is part of the Nature Communications initiative to facilitate training in peer review and to provide appropriate recognition for Early Career Researchers who co-review manuscripts.

Review: "Diversity-driven biochemical survey reveals dimeric structural origin of rubisco"

Reviewer Comments to Author:

Kehl et al. present a thorough investigation of Rubisco and Rubisco-like protein sequences to identity and classify deep branching groupings of these sequences. This approach has been conductive numerous times throughout the literature, usually with much smaller sequence sets, but the approach here is comprehensive and well developed. The group is highly skilled in these analyses and the tree matches my knowledge of the sequence relationships between the enzymes presented. Integrated analyses of kinetics, thermostability, and structure demonstrate that the dimeric architecture remains widespread, and functionally competent as a carboxylase in some deep branching clades. From the 102 putative Rubisco sequences examined only 18 could be recombinantly expressed in *E. coli*. From these 7 could be assigned RuBP carboxylase activity, and only 3 displayed a CO_2 fixation rate greater than 1 s^{-1}.

However, a Rubisco from one of the deep branching groups (the most distant to Form I Rubisco characterized?) displayed a CO2 fixation rate similar to some plant enzymes. They structurally characterize this enzyme to a high-resolution and resolve several novel secondary structural motifs arising from additional residues compared to other known Rubisco structures. The additional motifs are not interrogated in the context of the activity and it is not clear how widespread they are in nature. The authors call this the largest Rubisco described but it is much smaller than other Rubisco holoenzymes in that it is <150 kDa and many Rubisco are >500 kDa. Rather it is the Rubisco with the largest catalytic subunit, or longest RbcL described. Overall, the new data expands the structure and function understanding of Rubisco substantially and gets us closer to an understanding of how carbon fixation evolved.

In addition, the authors determine the oligomeric state and denaturation temperatures of the soluble enzymes and find these two features poorly correlated. Higher-order oligomerization is proposed to have evolved as an adaptive consequence to maintain L2 stability rather than as a functional role. This interpretation agrees with existing structure and kinetics studies that do not suggest to me any connection between oligomeric states and activity. Again, more progress is made towards a holistic understanding of Rubisco structure and function.

The authors interpret their data in the context of the new sequence topology of Rubisco determine a dimeric origin of Rubisco. I don't think anyone would doubt such an origin, as it is already obvious from the corpus of existing knowledge on Rubisco/RLP form and function. The study highlights the extreme amount of sequence diversity within the superfamily. They propose a new naming scheme for the whole family to accommodate this and future work, but I fear this will cause more confusion than it solves. Several concerns, particularly the first major point, should be addressed prior to publication as the analysis may fundamentally undermine the manuscript and I am unable to alleviate the concern easily from the material presented.

Major points:

- It is not overtly clear to me that what is presented as Rubisco are Rubisco at all. I randomly picked some of the sequences from the attached SI material and searched the genetic context of the genes. In some cases, including for some examples below (see pdf for table), the proposed *rbcL* gene is immediately preceded by a gene annotated for a S-methyl-5-thioribose-1-phosphate isomerase. This would surely make them RLPs that catalyze enolization reactions of this and similar substrates, as has been biochemically defined in the literature (Singh and Tabita, *J.*

Bacteriol. 2009). The authors have examined the operon context of all the Rubisco in the study, and therefore should use this information to clearly distinguish sequences/groups that are RLPs based on this operon context. Proximity to AMP-phosphorylase and ribose-1,5-bisphosphate isomerase would signal bona fide Rubisco involved in AMP metabolism (Sato et al. *Science*. 2007; Wrighton et al. *ISME*. 2016).

Protein ID	Species	Proposed Form	Genbank ref	Adjacent gene/protein	Adjacent annotation
NQU77245.1	Candidatus Falkowbacteria bacterium	VI	JABMQE010000023.1	NQU77244.1	S-methyl-5-thioribose-1-phosphate isomerase
MBI4990865.1	Candidatus Gottesmanbacteria bacterium	VI	JACRNZ010000050.1	MBI4990866.1	Hypothetical, but closest similarity is Methylthioribose-1-phosphate isomerase
MBI3413662.1	Candidatus Aenigmatarchaeota archaeon	V	JACPZZ010000034.1	MBI3413661.1	S-methyl-5-thioribose-1-phosphate isomerase
MFC7204168.1	Haloferax namakaokahaiae	V	JBHTAA010000005.1	MFC7204172.1	ribose 1,5-bisphosphate isomerase

- The proposed new naming system is flawed for several reasons. 1) The use of Greek and Latin to distinguish between two very different types looks similar and would be confusing (Ia and Iα for example). 2) The clear distinction that Form I Rubisco have small subunits is very useful and easy to communicate, but would be destroyed by the wholesale addition of many Rubisco that are proposed to be called Form I but lack them entirely. 3) It does not make things any clearer, and due to point 1) less clear, than the naming convention it is intended to replace.

  I sympathize with the authors on the challenge of categorizing the Rubisco superfamily, especially because of the diversity they have uncovered and will continue to describe. I do not have a perfect answer, however, I think they have an opportunity here to reclassify things in an organized way. A proposed classification for consideration: I agree with the distinction of RLPs as an RLP group as it is already a familiar nomenclature and distinguishes it from *bona fide* Rubisco. Form I Rubisco should be classified as containing a small subunit with Greek to denote sufficiently large and distinct groups following with a capitalized letter following the order of discovery and with a characterized representative species to contextualize the grouping. Form IF would make sense as a new group since it is distinct and likely represents early Form I enzymes. Perhaps groups without a representative species could be given a lower case as a proposed group placeholder until characterization is established (i.e Form If as already presented).

  This would then make the current Form I' as Form IIB (or whatever Greek most appropriate based on the publication date and the co-authors combined work on Form II Rubisco), rather than

Iβ – grounded by the characterized *P. breve*. *R. rubrum* would represent Form IIA, and there is plenty of room to include everything else and future discoveries from C – Z. Form III could follow a similar scheme grounded by *M. burtonii* and *T. kodakarensis* in at least two divisions. I suspect the authors will be displeased by this suggestion due to their remarkable discovery and naming of Form I', but it is the style of formatting that I think would be most widely adopted by the field and with room to grow.

- SEC-SAXS was performed in the presence or absence of CABP, which binds tightly to Rubisco active sites and has been documented to induce oligomerization from dimer (L2) to decamer (L10) (Alonso et al. JBC. 2017). Oligomerization from L2 to L10 was induced for two of the Rubisco in this study when CABP was present, but this not discussed. This is an important finding as the two Rubisco do not appear to contain, as best that I can surmise in the absence of explicit accessions, the domain insertion in RbcL biochemically described to mediate this process (Gunn et al. JBC. 2017). Clarity on this should be provided in the manuscript on the basis of their phylogenetic relationship to *M. burtonii* Rubisco and the current described mechanism of oligomerization.

Minor points:

- The accession codes for specific proteins described biochemically in the manuscript should be clearly documented in the methods or elsewhere so other researchers can confidently analyze/work with them.

- The phylogenetic context of the novel structural features identified in *P. syntrophicum* should be outlined. Is this something found in multiple lineages or isolated to a few enzymes in a single group?

- Intro: Paragraph 1. "only some of which involve carbon fixation". I'm not aware of any Rubisco in pathways that don't involve carbon fixation, its an intrinsic part of the enzyme's activity regardless of how minor the carbon fixed might be to the overall metabolism of the cell.

- Intro: Paragraph 2 The last sentence doesn't make sense to me. RLPs are either the retained ancestral form of proto-Rubisco or functionally diverged from an early proto-Rubisco. All RLPs are dimers and so are many Rubisco and it's the most simple organization of the functional form – so it ought to be the ancestral structure, unless a functional monomer can be observed. What quaternary structures are incomplete if all possible dimeric organizations have been listed and since those with RbcS are firmly locked as L8S8?

- Intro: Paragraph 5 Here and elsewhere in the manuscript the novel Rubisco is described as having a novel fold. My understanding is that protein domain folds are conserved arrangements of secondary structure. Rubisco has two folded domains an N-terminal domain of a ferredoxin fold and a C-terminal domain of a TIM-barrel fold. Mobile regions, minor insertions and deletions that don't disturb the broad architecture would not constitute a novel fold, and the superposition of the novel structure on 9RUB shows the overall fold is the same. I would avoid this description and instead use a precise articulation of the novel secondary structural elements.

- Since it is explicitly mentioned in the main text, it would be useful to list the Rubisco that were recombinantly expressed but found to be insoluble in the SI materials so that researchers can avoid these species in future work (see Davidi et al. *EMBO*. 2020 as an example).

- It is stated that "residues 342–353, which form a β-sheet that interacts with N-terminal residues 3–6 via an extended hydrogen-bonding network (**Figure 4f**)" but this interaction is not shown in the zoomed in panel depiction of this region.

- How is the denaturation temperature in SI Fig 5H assigned due to multiple similar peaks? The Tm values of Rubisco from the literature alongside the measurements from this study could be listed in an SI file.

- General formatting. Italicization of species names is absent in some sections (methods). Latin terms such as 'in vitro' and 'bona fide' should follow the journal style and be consistent throughout. Genes should be italicized (i.e. *rbcL*).

Best Regards,

Robbie Wilson
