# Supplementary material for: Diversity-driven biochemical survey reveals widespread dimerization throughout the rubisco superfamily
Source: Nat Commun. 2026 Jun 3;17:8069. doi: 10.1038/s41467-026-73982-5 (PMC13454268; doi:10.1038/s41467-026-73982-5)
Supplement: Supplementary file 3 [file 41467_2026_73982_MOESM1_ESM.pdf]

Diversity-driven biochemical survey reveals widespread dimerization throughout the rubisco superfamily

Supplementary Figures:

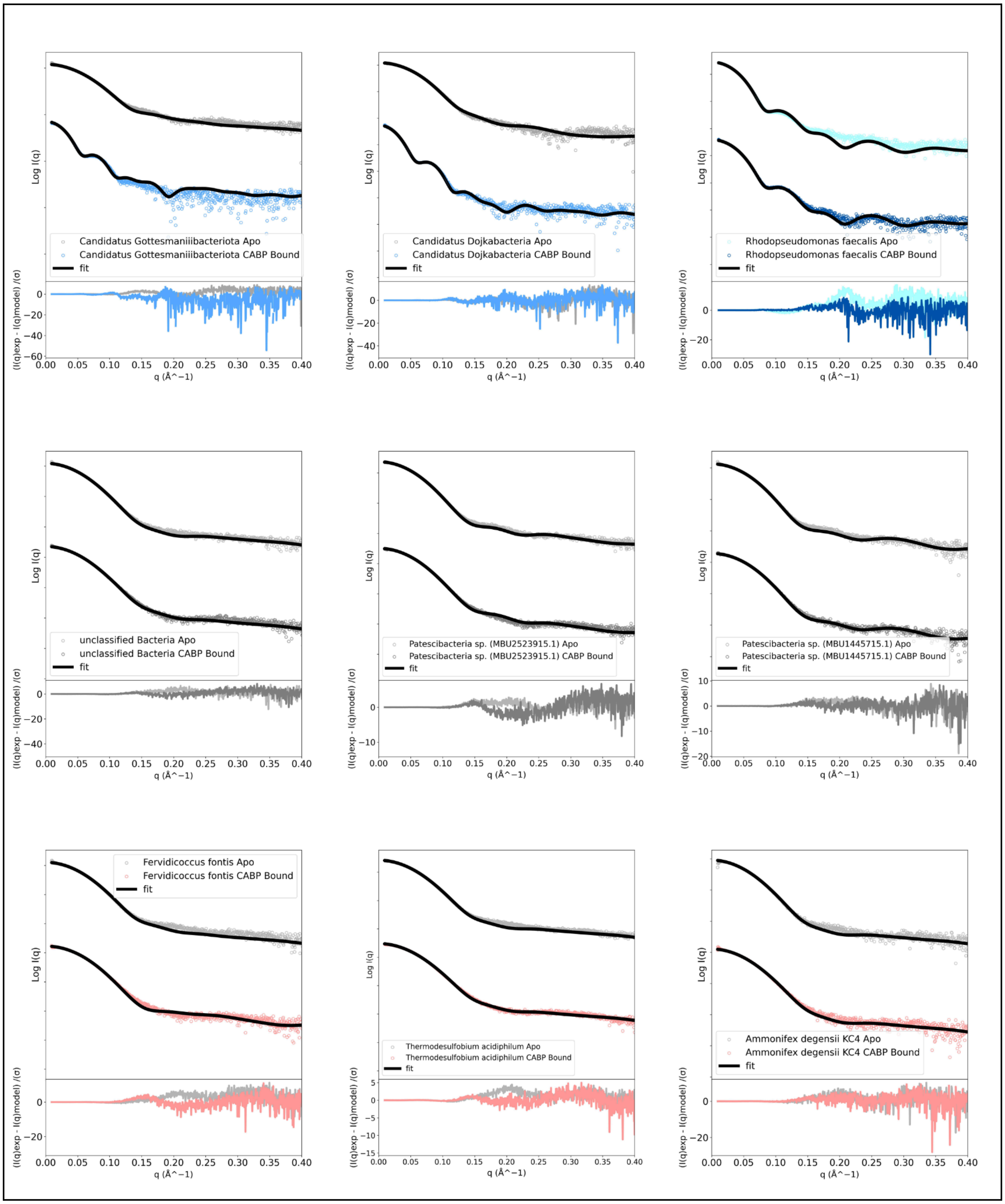

Supplementary Figure 1: **SAXS scattering profiles with fitted curves for rubiscos from *Candidatus Gottesmaniiibacteriota* through *Ammonifex degensii* KC4.** Experimental scattering data are shown as colored points, with best-fit theoretical curves overlaid (black lines). Residuals for each fit are plotted below the corresponding scattering profile in matching colors. Each panel is labeled with the source organism. Together, these data provide low-resolution structural validation across a phylogenetically diverse set of rubiscos. SAXS scattering profiles colored by clade; light blue: II-III(L2, L10), dark blue: II(L2, L4, L6), gray: unclassified(L2), salmon: Iβ(L2).

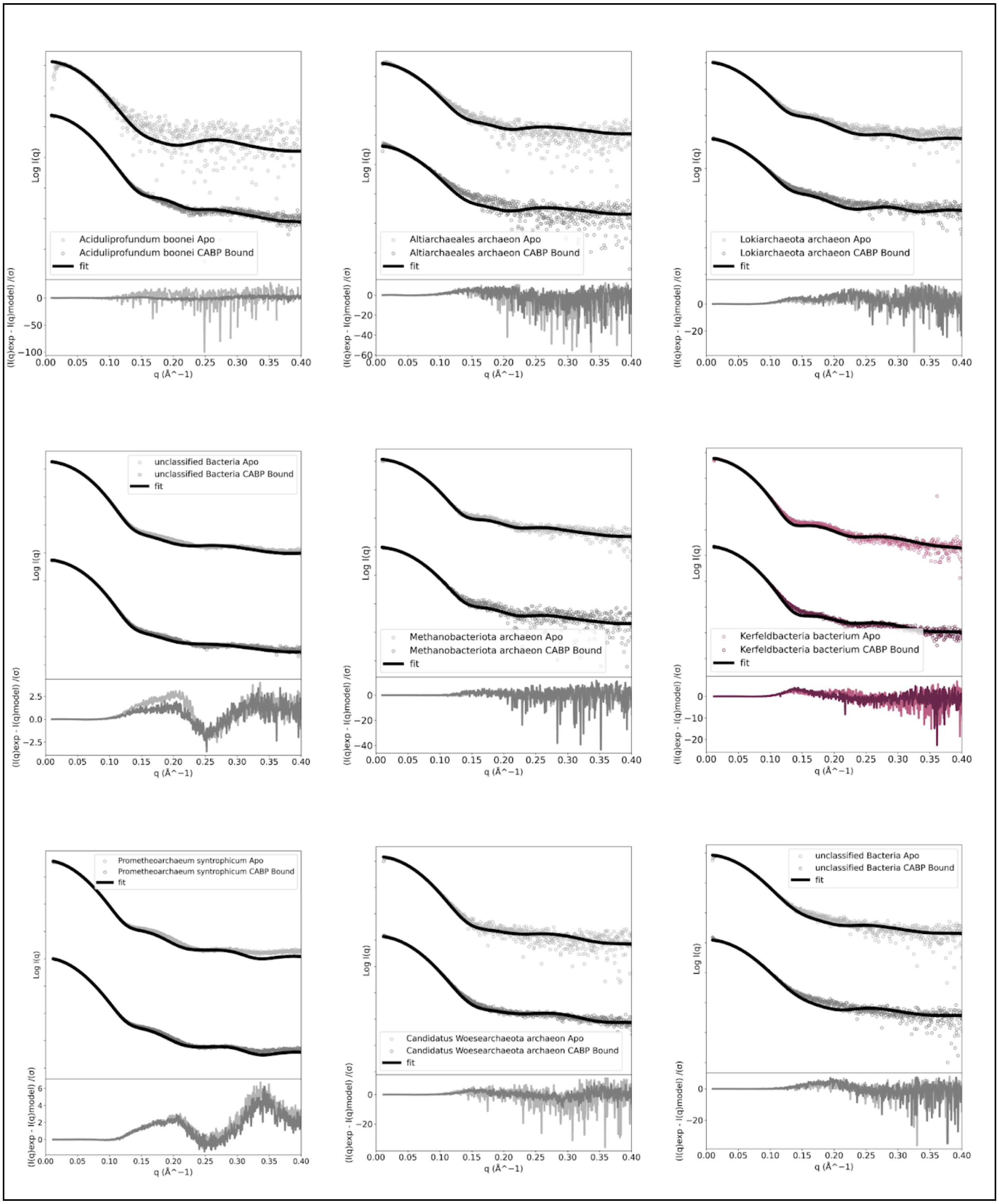

Supplementary Figure 2: **SAXS scattering profiles with fitted curves for rubiscos from** ***Aciduliprofundum boonei*** **through** ***unclassified Bacteria*****.** Experimental scattering data are shown as colored points, with best-fit theoretical curves overlaid (black lines). Residuals for each fit are plotted below the corresponding scattering profile in matching colors. Each panel is labeled with the source organism. Together, these data provide low-resolution structural

validation across a phylogenetically diverse set of rubiscos. SAXS scattering profiles colored by clade; gray: unclassified(L2), dark purple: III-Like(L2).

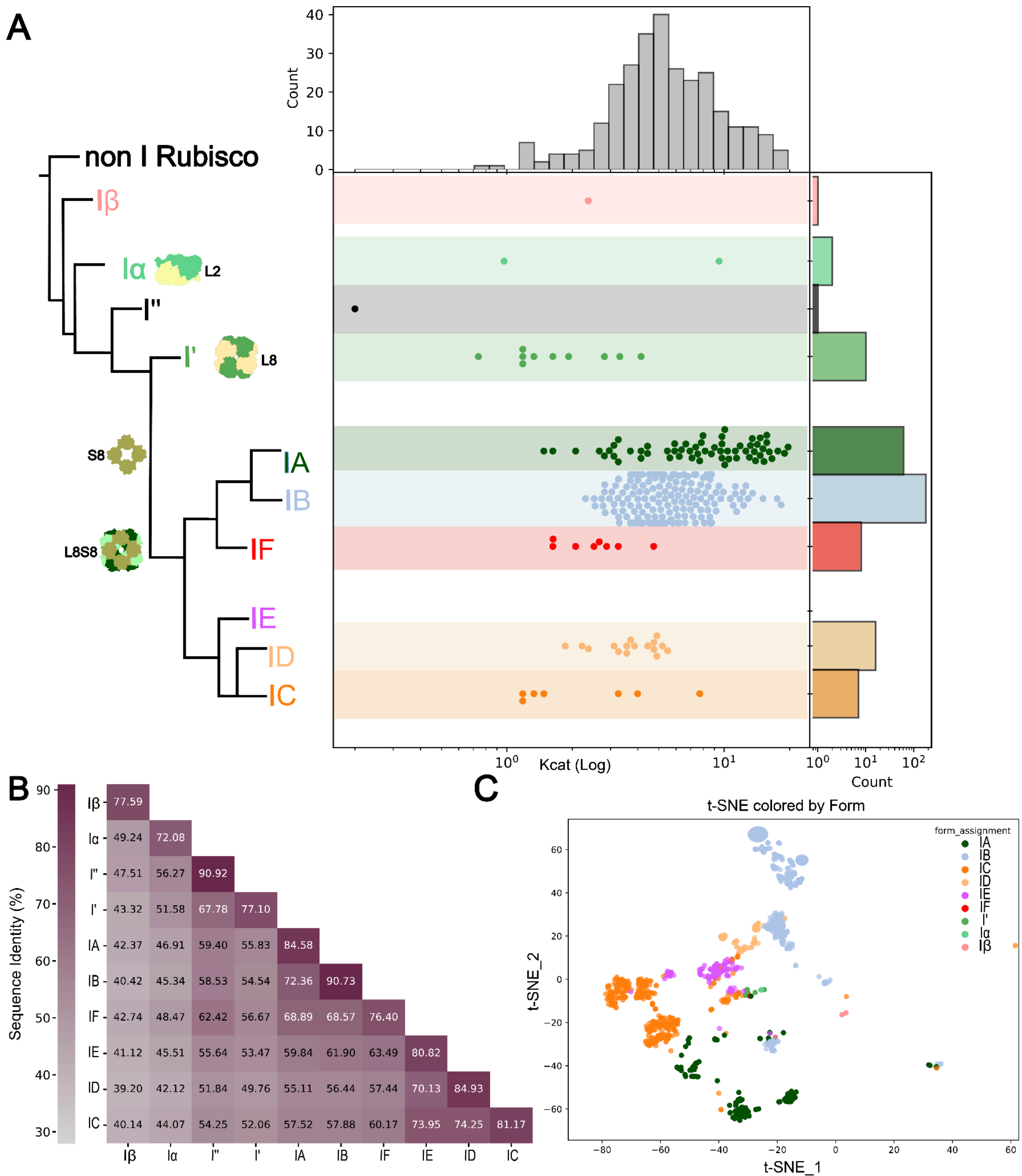

Supplementary Figure 3: Phylogenetic tree schematic of the rubisco form I clade; a simplified tree showing oligomeric and kinetic distribution. **A:** Known clades are labeled

with the new nomenclature. Clades or clusters with k_{catC} (Log) measurement in line with the tree. Top histogram shows the count and distribution of k_{catC}(Log) measurements by k_{catC}, the right histogram shows the count and distribution of k_{catC}(Log) measurements by subform. **B:** pairwise sequence identity heatmap. Sequence identity is shown for all form I subforms of rubisco with numeric values and color; deep purple: >90% sequence identity, gray: ~30% sequence identity. **C:** t-SNE of rubisco form I subform sequences colored by subform; dark blue: 1A (L8S8), light blue: 1B(L8S8), dark orange: 1EC(L8S8), light orange: 1D(L8S8), purple: 1E(L8S8), red: 1F(L8S8), dark green: I'(L8), light green. Iα(L8), samon: Iβ(L2), black I''(L2). PDBs used in this figure are as follows form I: 1RBL, 6URA.

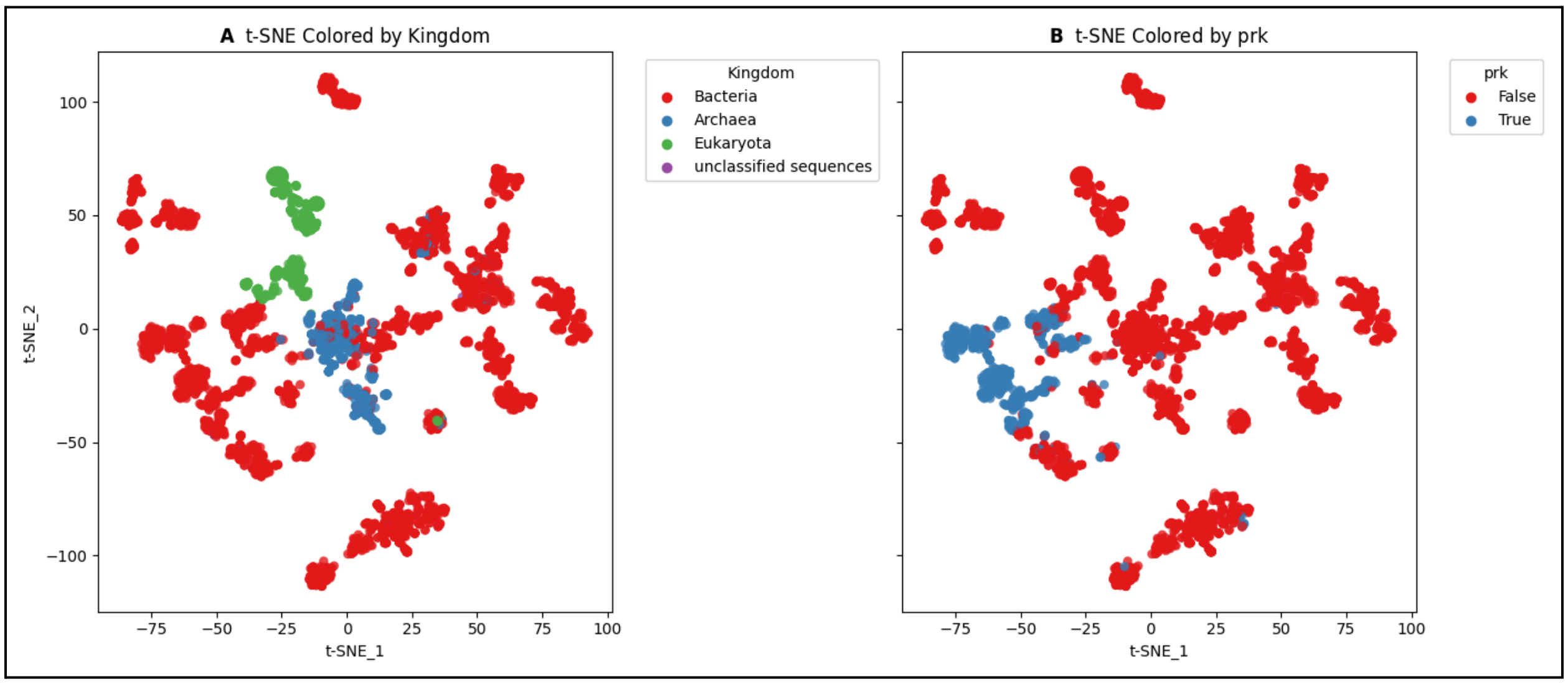

Supplementary Figure 4: **TSNE color-coded by domain of life and prk .** t-SNE of all rubisco, including RLPs colored by kingdom (A); Bacteria: red, Archaea: blue, Eukaryota: green, unclassified: purple, and presence of PRK (B); False: red, True: blue

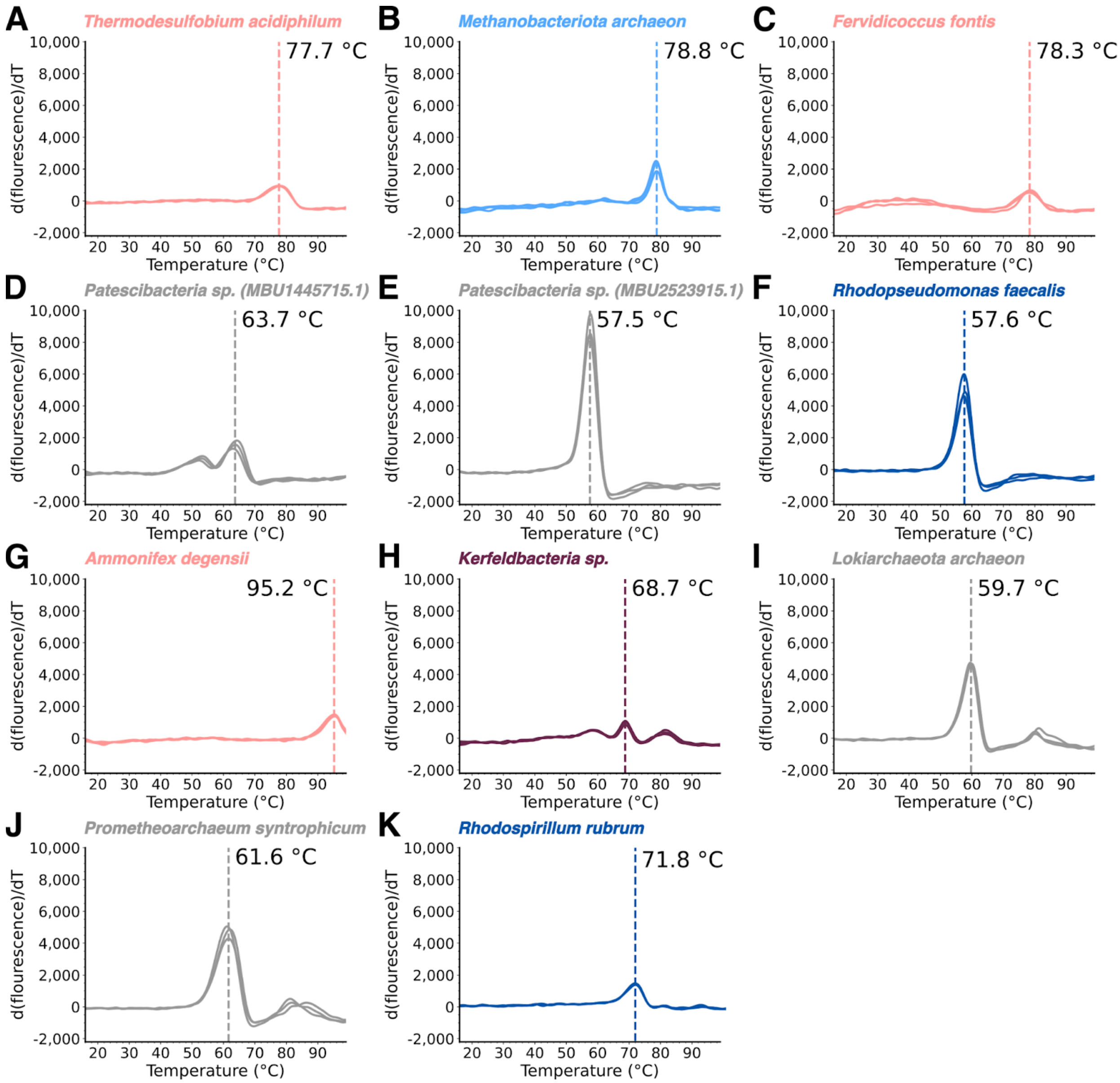

Supplementary Figure 5: Melting curves for rubiscos described in Supplementary table 3. **A-K)** Protein thermal shift assay data with annotated melting temperatures (T_m) of newly characterized rubisco dimers. Reported T_m values represent the average obtained from three technical replicates. Melting curves colored by clade; salmon: Iβ(L2), light blue: II-III(L2, L10), gray: unclassified(L2), dark blue: II(L2, L4, L6), dark purple: III-Like(L2).

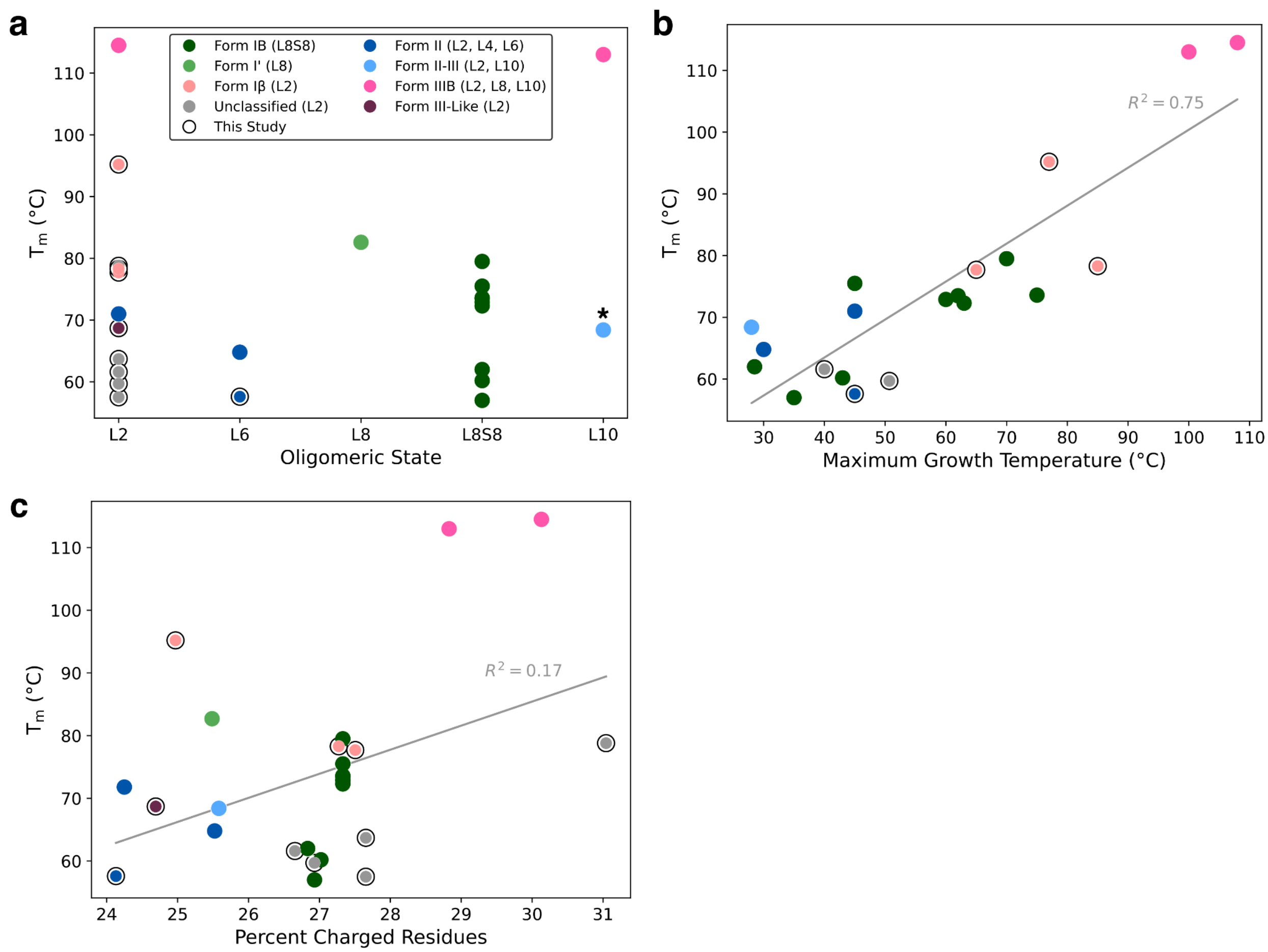

Supplementary Figure 6. Determinants of rubisco thermal stability: weak influence of oligomeric state and charge, strong correlation with source organism growth temperature. **A)** T_m plotted against rubisco's oligomeric state reveals minimal correlation. **B)** T_m of rubisco plotted against the maximum growth temperature of its source organism. Positive correlation between T_m and maximum growth temperature suggests that evolution of thermal stability in rubiscos was driven by the temperature of its source organism's habitat as opposed to oligomeric state. Asterisk: *Methanococcoides burtonii* can assemble into both dimer and decamer based on the presence of substrate, inhibitors or divalent metal ions. Plot reports T_m of *Methanococcoides burtonii* as decamer. **C)** T_m plotted against percent charged residues reveals minimal correlation. Scatter plot colored by clade; dark green: IB(L8S8), light green: I'(L8), salmon: Iβ(L2), gray: unclassified(L2), dark blue: II(L2, L4, L6), light blue: II-III(L2, L10), pink: IIIB(L2, L8, L10), dark purple: III-Like(L2). Outlined points indicate T_m collected within this study.

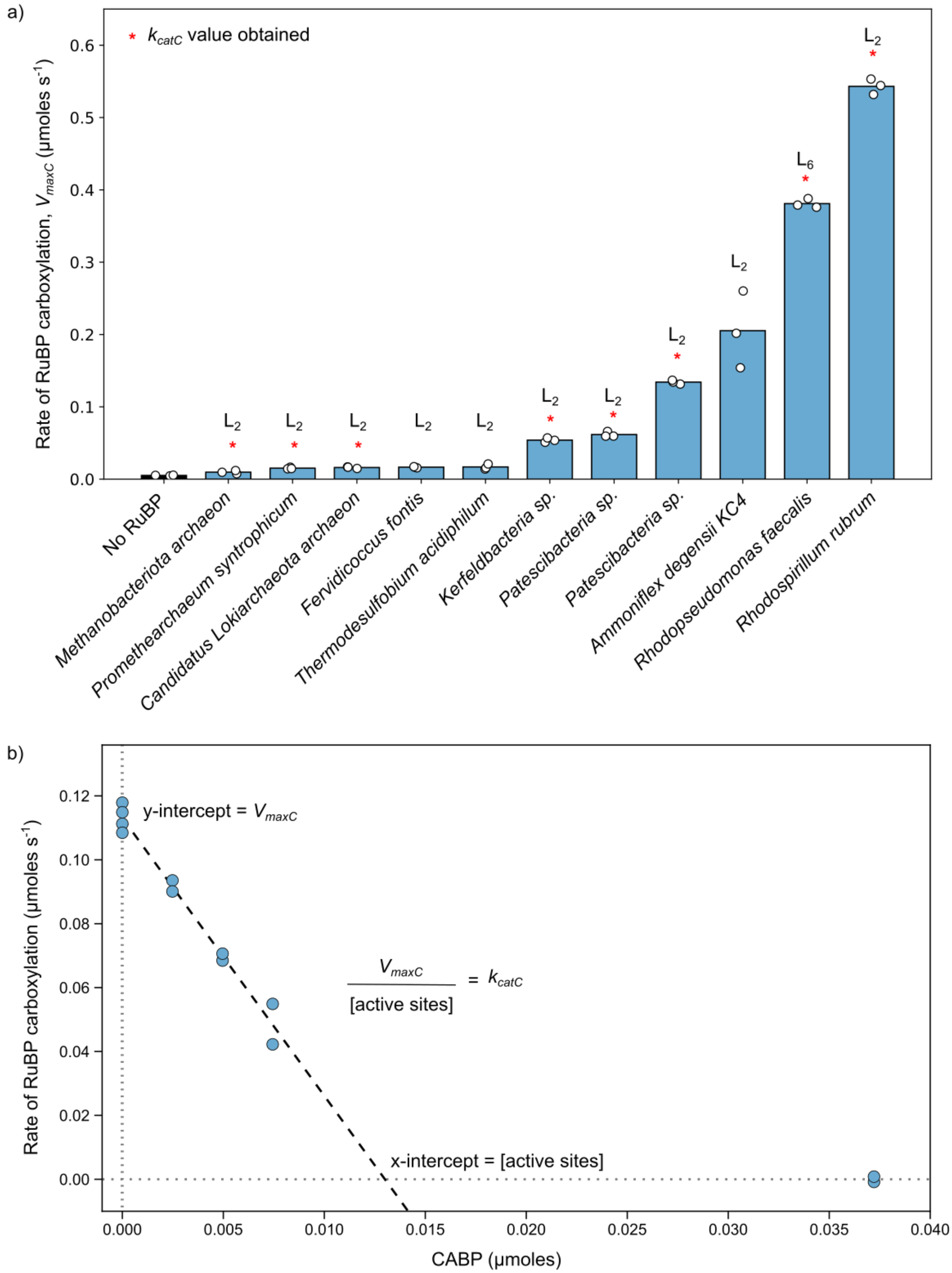

a)
* k_{catC} value obtained
Rate of RuBP carboxylation, V_{maxC} (µmoles s^{-1})
0.0
0.1
0.2
0.3
0.4
0.5
0.6
L_2
L_6
No RuBP
Methanobacteriota archaeon
Promethearchaeum syntrophicum
Candidatus Lokiarchaeota archaeon
Fervidicoccus fontis
Thermodesulfobium acidiphilum
Kerfeldbacteria sp.
Patescibacteria sp.
Patescibacteria sp.
Ammonifiex degensii KC4
Rhodopseudomonas faecalis
Rhodospirillum rubrum
b)
y-intercept = V_{maxC}
$\frac{V_{maxC}}{[\text{active sites}]} = k_{catC}$
x-intercept = [active sites]
Rate of RuBP carboxylation (µmoles s^{-1})
0.00
0.02
0.04
0.06
0.08
0.10
0.12
0.000
0.005
0.010
0.015
0.020
0.025
0.030
0.035
0.040
CABP (µmoles)

Supplementary Figure 7: **Enzymatic Activity and Carboxylation Kinetics of Rubisco. a)** The carboxylation activity of all active rubisco sequences. Those for which carboxylation turnover number, k_{catC}, values could be determined are marked with a red *; n=3 technical replicates. **b)** A CABP-titration assay determines k_{catC}. The rate of rubisco carboxylation is plotted as a function of CABP concentration. The x-intercept gives the concentration of rubisco active sites, [active sites], while the y-intercept gives the reaction rate without CABP inhibition, V_{max}. Here, the k_{catC} for carboxylation of *Rhodospirillum rubrum* ≈ 9 s^{-1}. CABP-containing reactions, n=2, whereas the CABP-free reaction,
n=4, technical replicates. Dark blue represents the negative control with no RUBP, light blue represents the samples containing both enzyme and substrate.

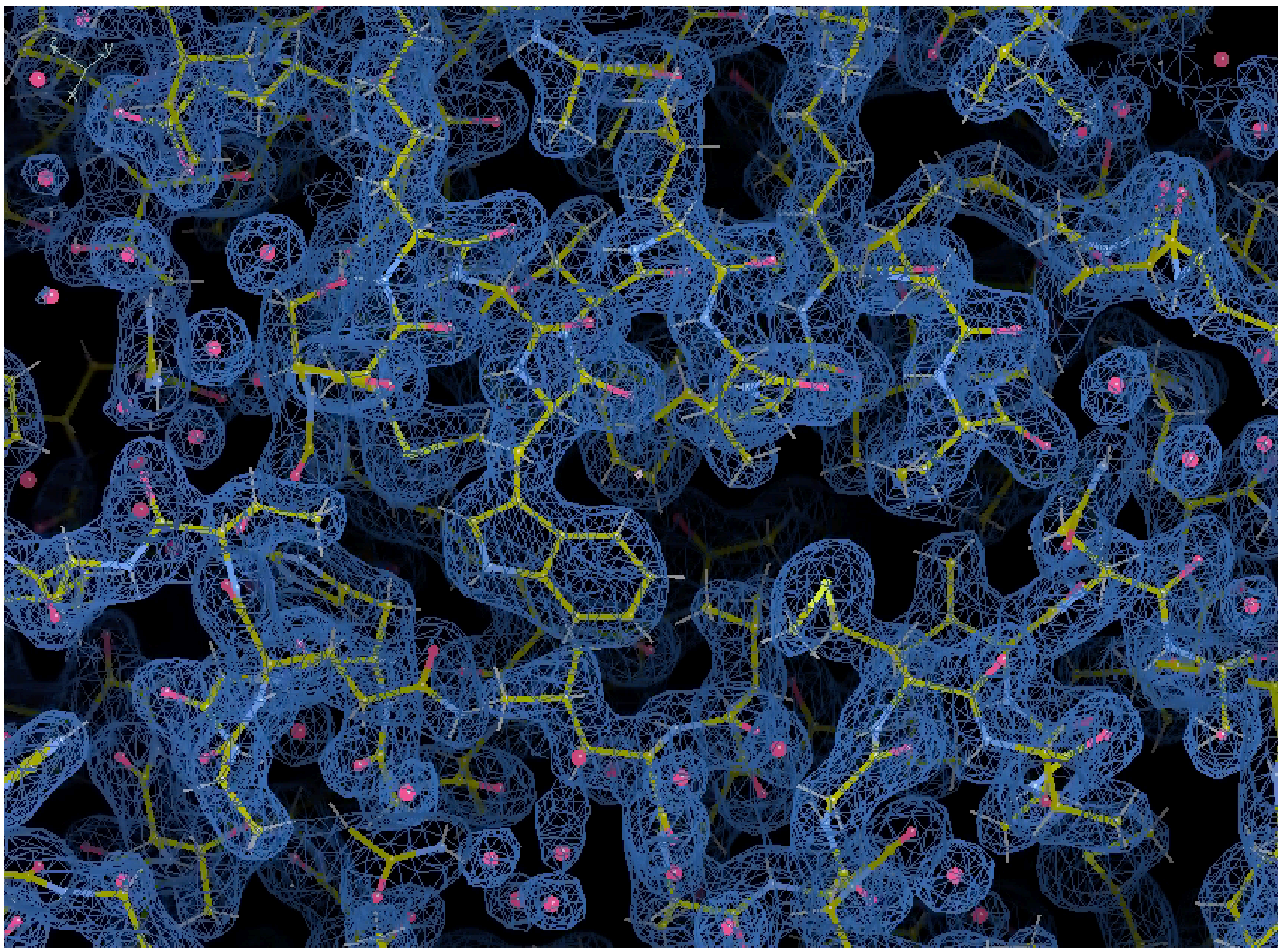

Supplementary Figure 8. 2mFo-DFc electron density map contoured at 1.5 sigma level for the crystal structure of the dimeric rubisco from Prometheoarchaeum syntrophicum (PsRuB)

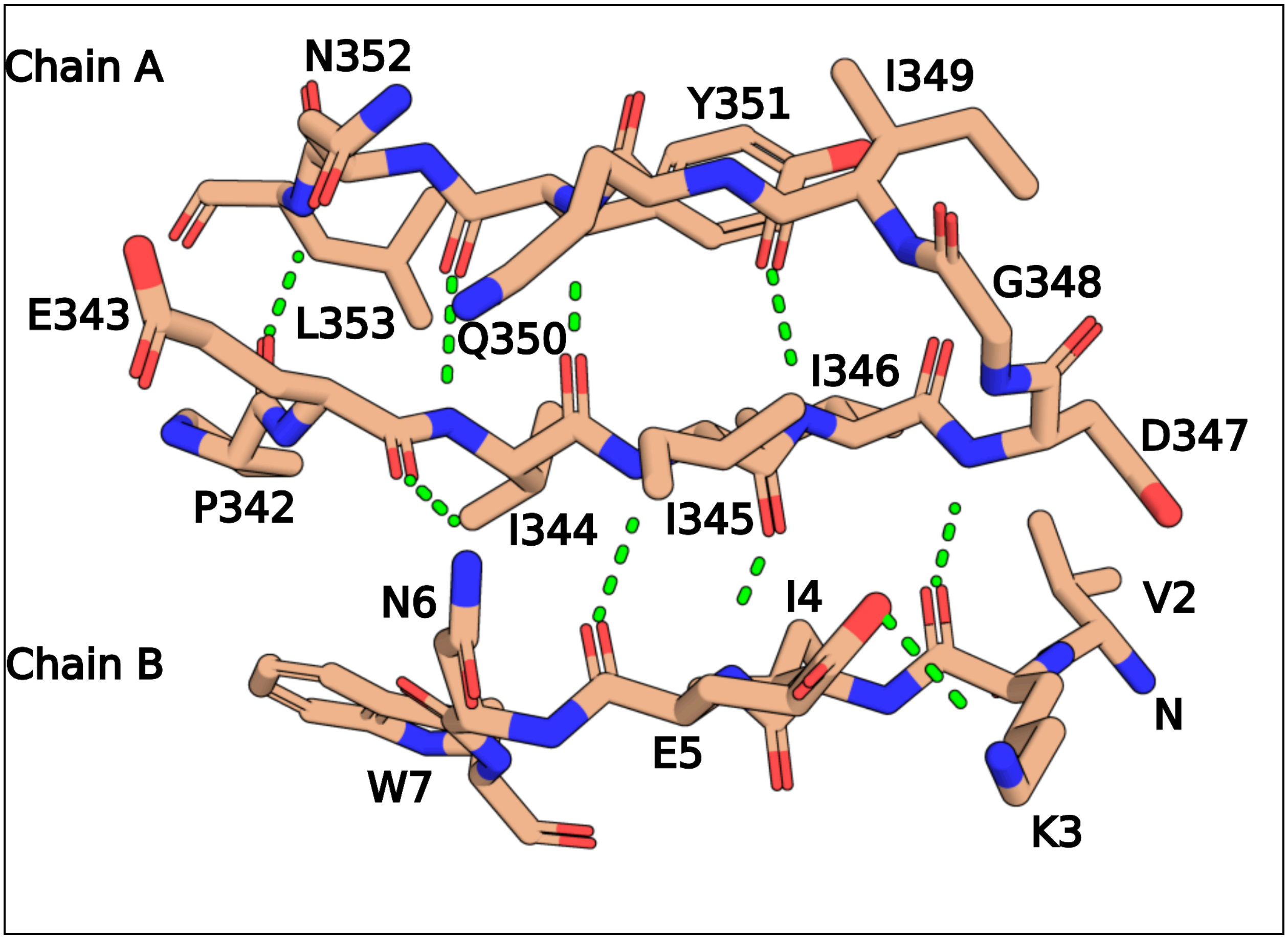

Supplementary Figure 9: Residues 342–353, which form a β sheet that interacts with N-terminal residues 3–6 via an extended hydrogen bonding network in *Prometheoarchaeum syntrophicum* rubisco. Orange is used to denote carbon, blue: nitrogen, red: oxygen, green: hydrogen bond.

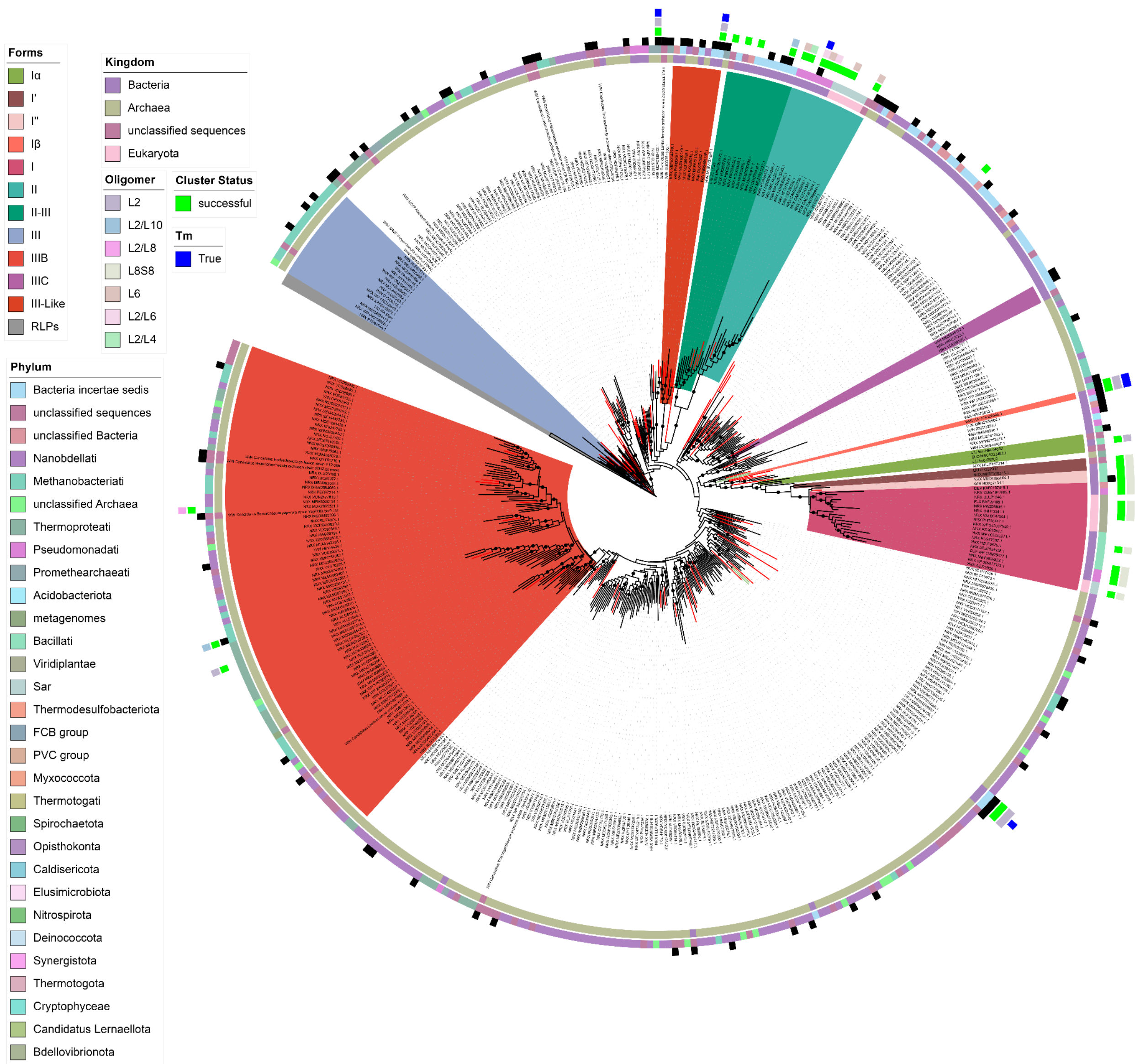

Supplementary Figure 10: Comprehensive phylogenetic tree highlighting rubisco clusters characterized for activity and oligomeric state. Green highlights mark clusters previously characterized for enzymatic activity, while black bars indicate clusters analyzed in this study. Additional annotations denote oligomeric state and thermal stability (Tm).

Supplementary Tables:

Supplementary Table 1: SEC-SAXS oligomeric state determination CABP bound

Accession	Simple Scattering Code	Organism	MW(kDa) Sequence	MW (kDa)	Rg (Å)	χ^2	Oligomeric state
OGG30932.1	*XSF2W1AA*	*Candidatus Gottesmaniib*	*54*	548	58	4.4	L10

		acteriota					
KKP92911.1	*XSZNZ3J0*	*Candidatus Dojkabacteria*	*52*	-	56	2.0	L10
WP_110780429.1	*XSQSTHGE*	*Rhodopseudomonas faecalis*	*50*	261	43	1.3	L6
RKZ25299.1	*XSBQGHPR*	*unclassified bacteria*	*45*	71	29	2.6	L2
MBU2523915.1	*XSGT6MLN*	*Patescibacteria sp. (MBU2523915.1)*	*50*	79	29	2.4	L2
MBU1445715.1	*XSKDNJSI*	*Patescibacteria sp. (MBU1445715.1)*	*48*	75	29	1.1	L2
PMB76193.1	*XSXPYDB3*	*Fervidicoccus fontis*	*47*	73	28	2.1	L2
WP_108308545.1	*XS93BFEP*	*Thermodesulfobium acidiphilum*	*47*	73	28	4.1	L2
ACX52946.1	*XSZQWWUB*	*Ammonifex degensii KC4*	*45*	-	28	1.3	L2
WP_008086508.1	*XSVZ5KE8*	*Aciduliprofundum boonei*	*47*	58	26	1.0	L2
HIE34177.1	*XS895INW*	*Altiarchaeales archaeon*	*45*	77	37	4.0	L2
Candidatus_Lokiarchaeota_archaeon_strain_HM5_B55	*XSPQVMS8*	*Lokiarchaeota archaeon*	*62*	97	35	6.9	L2
HEU31633.1	*XS2QFXBK*	*unclassified bacteria*	*43*	77	29	7.8	L2

HDR53286.1	*XSFWYF0V*	*Methanobacteriota archaeon*	*47*	74	28	1.1	L2
MBI5465798.1	*XSULRK2B*	*Kerfeldbacteria bacterium*	*53*	82	29	8.5	L2
WP_147662334.1	*XSGMMWKQ*	*Prometheoarchaeum syntrophicum*	*62*	99	32	19.8	L2
PIU30467.1	*XSMGG3DP*	*Candidatus* "Woesearchaeota archaeon CG07_land_8_20_14_0_80_44_2"	*47*	75	29	4.3	L2
NOY14888.1	*XSCJOKGI*	*unclassified bacteria*	*46*	73	30	1.4	L2

Supplementary Table 2: SEC-SAXS oligomeric state determination unbound

Accession	Simple Scattering Code	Organism	MW(kDa) Sequence	MW(kDa) SAXS	Rg (Å)	χ^2	Oligomeric state
OGG30932.1	*XSF2W1AA*	*Candidatus Gottesmaniibacteriota*	*54*	87	31	3.9	L2
KKP92911.1	*XSZTP5RF*	*Candidatus Dojkabacteria*	*52*	87	30	2.2	L2
WP.110780429.1	*XSPVEHHK*	*Rhodopseudomonas faecalis*	*50*	262	43	6.4	L6
RKZ25299.1	*XSKWLUDQ*	*unclassified Bacteria*	*45*	74	29	2.5	L2
MBU2523915.1	*XSPPBS9X*	*Patescibacteria sp. (MBU252391*	*50*	84	30	2.5	L2

		5.1)					
MBU1445715.1	*XSQC2P66*	*Patescibacteria sp. (*MBU1445715.1*)*	*48*	-	29	1.7	L2
PMB76193.1	*XSQ5HCCW*	*Fervidicoccus fontis*	*47*	76	29	4.2	L2
WP.108308545.1	*XSDPZBFI*	*Thermodesulfobium acidiphilum*	*47*	76	29	6.4	L2
ACX52946.1	*XSE1JAJX*	*Ammonifex degensii KC4*	*45*	76	29	1.9	L2
WP.008086508.1	*XSN0OSDA*	*Aciduliprofundum boonei*	*47*	75	31	0.9	L2
HIE34177.1	*XSQIRSSH*	*Altiarchaeales archaeon*	*45*	85	36	4.8	L2
Candidatus_Lokiarchaeota_archaeon_strain_HM5_B55	*XSJGHHBY*	*Lokiarchaeota archaeon*	*62*	100	34	4.6	L2
HEU31633.1	*XSEGORYK*	*unclassified bacteria*	*43*	77	29	20	L2
HDR53286.1	*XSOXNSZO*	*Methanobacteriota archaeon*	*47*	79	28	1.6	L2
MBI5465798.1	*XSPR2OHY*	*Kerfeldbacteria bacterium*	*53*	88	30	5.2	L2
WP.147662334.1	*XSANCTQG*	*Prometheoarchaeum syntrophicum*	*62*	101	32	37	L2
PIU30467.1	*XSDNKW9Y*	*Candidatus “*Woesearchaeota archaeon CG07_land_8_20_14_0_80_44_2”	*47*	78	30	1.4	L2

NOY14888.1	*XSY0ANCM*	*unclassified bacteria*	*46*	75	29	1.8	L2

Supplementary Table 3: Carboxylation activity of phylogenetically diverse rubiscos

Accession	Organism	form	k_{catC}	k_{catC}SD
Candidatus_Lokiarchaeota_archaeon_strain_HM5_B55	*Lokiarchaeota archaeon*	unclassified	0.3	0.040
WP_147662334.1	*Prometheoarchaeum syntrophicum*	unclassified	2.4	0.005
MBU1445715.1	*Patescibacteria sp. (MBU1445715.1)*	unclassified	0.6	0.049
MBU2523915.1	*Patescibacteria sp. (MBU2523915.1)*	unclassified	2.4	0.332
HDR53286.1	*Methanobacteriota archaeon*	unclassified	0.3	0.073
MBI5465798.1	*Kerfeldbacteria sp.*	III-LIKE	0.7	0.066
WP110780429.1	*Rhodopseudomonas faecalis*	II	9.6	0.824
WP_073240254.1	*Rhodospirillum rubrum*	II	9.8	0.458

Supplementary Table 4: Crystallographic statistics for X-rays data collection and structure refinement.

	**PsRuB**
Data collection	
Space group	P 1
Unit-Cell parameters (Å)	69.55 82.7 113.3 108.17 97.61 103.23
Resolution range (Å)	46.38 - 1.9
Total reflections	682393 (28461)
Unique reflections	161119 (13462)
R_{merge} (%)	0.121 (1.15)
$I/\sigma I$	6.3 (0.9)
Wilson B-factor	22.7
Completeness (%)	99.5 (98.1)
Redundancy	3.8 (3.2)
$CC_{1/2}$	0.994 (0.311)
Refinement	
Resolution range (Å)	46.38 - 1.9 (1.93 - 1.9)
Reflections used in refinement	161102 (13462)

Reflections used for R_{free}	1809 (151)
R_{work}	0.1755 (0.3361)
R_{free}	0.2051 (0.3599)
No. atoms	
Proteins	17437
Ligands/ion	40
Water	1400
RMS from ideal geometry	
Bond lengths (Å)	0.007
Bond angles (º)	0.71
Average B-factor	42.47
Macromolecules	42.87
Ligands	37.48
Solvent	37.60
Ramachandran favored (%)	95.99
Ramachandran allowed (%)	3.74
Ramachandran outliers (%)	0.27
Clashscore	3.36

[#] Values in parentheses are for the highest-resolution shell.
