# Supplementary material for: Diversity-driven biochemical survey reveals widespread dimerization throughout the rubisco superfamily
Source: Nat Commun. 2026 Jun 3;17:8069. doi: 10.1038/s41467-026-73982-5 (PMC13454268; doi:10.1038/s41467-026-73982-5)
Supplement: Supplementary file 4 [file 41467_2026_73982_MOESM2_ESM.pdf]

Description of Additional Supplementary Files

Supplementary Data 1 — Centroid metadata for the rubisco superfamily phylogeny. Per-centroid metadata for the 1,859 sequence clusters (65% amino-acid identity threshold) used to reconstruct the maximum-likelihood phylogeny of the rubisco superfamily shown in Figure 1a. Columns include the full and brief sequence headers, an indicator for sequences flagged for biochemical characterization in this study, cluster characterization status (successful, attempted, or neither), cluster ID, reconciled NCBI taxonomic lineage, assigned clade name, centroid amino-acid sequence and length, and branch length on the inferred tree.

Supplementary Data 2 — Centroid metadata for the form I subtree. Per-centroid metadata for the 1,528 sequence clusters (90% amino-acid identity threshold) used to construct the more deeply sampled form I subtree shown in Figure 1b. Columns include sequence headers, characterization flag, cluster ID, reconciled NCBI taxonomic lineage, assigned form I subform (IA, IB, IC, ID, IE, IF, I′, Iα, Iβ, I″, or UNK), centroid amino-acid sequence, and length.

Supplementary Data 3 — Compiled rubisco kinetic dataset. Carboxylation turnover rates (*k*~catC~) for 448 cluster–member sequence pairings spanning forms I, II, II-III, IIIB, Iα, Iβ, I′, I″, III-Like, and unclassified clades, compiled from this study and the published literature. Columns include cluster ID and size, centroid and member sequence headers, source organism, assigned form, large- and small-subunit amino-acid sequences, lab-corrected carboxylation rate, source citation(s), and taxonomic kingdom. Underlies the kinetic distribution analysis in Figure 3a and Supplementary Figure 8.

Supplementary Data 4 — Long-format pairwise sequence identity table. All-versus-all pairwise amino-acid sequence identities for the representative rubisco set used in the phylogenetic reconstruction (~274,911 sequence pairs). Columns include the two compared sequence identifiers, percent identity calculated with and without gaps, the number of aligned residues compared in each case, and the assigned clade and subform for each sequence.

Supplementary Data 5 — Genomic context and t-SNE metadata. Per-locus metadata for the 5,774 rubisco genomic neighborhoods analyzed by t-SNE in Figure 3c and Supplementary Figure 2. Columns include NCBI rubisco protein and scaffold accessions, source organism, NCBI taxonomic lineage and domain, assigned clade and subform, cluster characterization status, presence/absence flags for the rubisco small subunit (*RbcS*) and phosphoribulokinase (*prk*), and t-SNE coordinates (tsne_dim_1, tsne_dim_2).

Supplementary Data 6 — Master rubisco sequence library. Full set of 87,030 rubisco-superfamily and rubisco-like protein amino-acid sequences assembled for this study. The dataset field indicates the source of each sequence: NRX (NCBI non-redundant database), ENV (NCBI env_nr database), and per-publication tags identifying sequences drawn from prior kinetic studies. Each entry includes the cleaned and full sequence headers, sequence length, amino-acid sequence, and assigned clade and subform under our updated classification.

Supplementary Data 7 — Solubility screen results. Outcomes of the heterologous expression and solubility screen for the diverse rubiscos selected for biochemical characterization in this study. Columns include the NCBI accession, amino-acid sequence, sequence length, and a Boolean indicator of whether soluble protein was recovered after purification from *E. coli*.

Supplementary Data 8 — Thermostability and growth-temperature dataset. Melting temperatures (Tm) and related thermal metrics for 35 rubiscos, comprising 11 Tm values measured in this study together with literature-derived Tm, T50, and maximum growth temperature values. Columns include source organism, NCBI accession, amino-acid sequence, assigned form, Tm and Tm standard deviation, Tm source citation and link, host maximum growth temperature with source and link, and T50 with standard deviation, source, and link where available. Underlies the thermostability analyses in Table 2 and Supplementary Figures 6 and 7.
